# Recovery Kinetics and Postoperative Outcomes of Propofol‐Based Total Intravenous Anesthesia Versus Sevoflurane in Ambulatory Surgery: A Systematic Review and Meta‐Analysis of Randomized Studies

**DOI:** 10.1155/anrp/2531080

**Published:** 2026-10-01

**Authors:** Ivan Šitum, Roberto Giurazza, Martina Perić Šitum, Ante Erceg, Slobodan Mihaljević, Marco Rispoli, Francesco Coppolino, Zoran Karlović

**Affiliations:** ^1^ Department of Anesthesiology and ICU University Hospital Centre Zagreb Zagreb Croatia kbc-zagreb.hr; ^2^ University of Zagreb School of Dental Medicine Zagreb Croatia; ^3^ Department of Critical Care AORN Ospedali Dei Colli Naples Italy nosm.ca; ^4^ Department of Woman, Child, General and Specialized Surgery University of Campania “Luigi Vanvitelli” Naples Italy unina2.it; ^5^ Department of Neurology Dubrava University Hospital Zagreb Croatia; ^6^ Department of Dental Medicine University Hospital Centre Zagreb Zagreb Croatia kbc-zagreb.hr

**Keywords:** ambulatory surgery, meta-analysis, postoperative nausea and vomiting, postoperative pain, propofol, sevoflurane, total intravenous anesthesia

## Abstract

**Background:**

Optimal anesthetic selection is critical for recovery and patient outcomes in ambulatory surgery. This systematic review and meta‐analysis compares propofol‐based total intravenous anesthesia (TIVA) with sevoflurane in adults undergoing outpatient procedures, focusing on randomized evidence published since 2014 to reflect contemporary clinical practice.

**Methods:**

We performed a PRISMA‐compliant, random‐effects meta‐analysis of 15 randomized controlled trials (*n* = 1345 randomized participants), published between 2014 and 2025, comparing propofol TIVA with sevoflurane in adults undergoing ambulatory surgery. Primary outcomes were readiness for discharge, postanesthesia care unit (PACU) length of stay (LOS), and emergence time. Secondary outcomes included postoperative nausea and vomiting (PONV) and pain scores, requirement for rescue antiemetics and analgesics, unplanned hospital admissions, and adverse events. All‐cause mortality was prespecified for extraction but was not reported in the included trials (PROSPERO: CRD420251151311).

**Results:**

Fifteen RCTs were included. For the primary outcomes, discharge readiness was shorter with TIVA in a single core trial (not a pooled meta‐analytic estimate) (mean difference [MD] −5.8 min; 95% confidence interval [CI] −10.63 to −0.97; *p* = 0.019), although no significant differences were observed for PACU LOS (MD ‐1.3 min; 95% CI −7.3 to 4.8; *p* = 0.56). Emergence times were not pooled due to extreme heterogeneity between studies. Regarding secondary outcomes, TIVA was associated with a lower risk of PONV (risk ratio 0.67; 95% CI 0.47–0.96; absolute risk difference −0.11; approximate number needed to treat 9), with attenuation toward the null under trim‐and‐fill, and with lower postoperative pain scores (standardized mean difference [SMD] −0.30; among VAS 0–10 trials, MD −0.29 points). Mortality was not reported in any of the included trials.

**Conclusions:**

Contemporary randomized evidence suggests that propofol TIVA may reduce PONV and yield a small reduction in postoperative pain scores. Evidence regarding discharge readiness, PACU LOS, and emergence is limited or inconsistent and does not support a general claim of faster early recovery with either technique. Anesthetic choice should balance these symptomatic effects against patient factors and institutional resources.

**Summary**
• Optimal anesthetic selection is critical for recovery in ambulatory surgery. This systematic review and meta‐analysis compared propofol‐based total intravenous anesthesia with sevoflurane in adults undergoing outpatient procedures, including 15 randomized controlled trials (*n* = 1345 randomized participants) published between 2014 and 2025, identified through a comprehensive search of major databases. Random‐effects models with the Hartung–Knapp–Sidik–Jonkman method were used for quantitative synthesis. For time‐based recovery measures that are typically log‐normally distributed, we additionally analyzed ratios of means on the log scale. Certainty of evidence was graded according to the GRADE framework.
• Time to discharge readiness was available from a single core trial (Kim et al., *n* = 99) and is reported as an individual‐study result, not a pooled random‐effects estimate (mean difference −5.8 min; 95% confidence interval −10.63 to −0.97; *p* = 0.019). Postanesthesia care unit length of stay did not differ significantly (mean difference −1.26 min; 95% confidence interval −7.34 to 4.83; *p* = 0.56) (ratio of means 0.92; 95% confidence interval 0.70–1.21), with substantial residual heterogeneity. Emergence time was analyzed as ratios of means but remained extremely heterogeneous (*I* ^2^ ≈ 98%) and is not interpreted as a clinically meaningful pooled effect. Propofol‐based total intravenous anesthesia significantly reduced postoperative nausea and vomiting incidence compared to sevoflurane (risk ratio 0.67; 95% confidence interval 0.47–0.96; absolute risk difference −0.11; approximate NNT 9), with attenuation toward the null under trim‐and‐fill sensitivity analysis and was associated with lower postoperative pain scores (standardized mean difference −0.30; 95% confidence interval −0.51 to −0.09; among VAS 0–10 trials, mean difference −0.29 points). Mortality was not reported in any included trial.
• Contemporary randomized evidence suggests that propofol TIVA may reduce PONV and produce a small reduction in early postoperative pain scores, whereas evidence on discharge readiness, PACU stay, and emergence is limited or inconsistent. Anesthetic choice should be individualized according to PONV risk, available resources, and local pathways. This review cannot assess rare major safety outcomes such as mortality.

## 1. Introduction

Patient expectations in outpatient surgery have evolved significantly over the past two decades, driven by the progressive migration of procedures historically requiring inpatient admission into the ambulatory setting, as well as by a parallel rise in patient‐valued outcome priorities. Ambulatory surgery now accounts for approximately 60%–70% of elective procedures in developed nations [1, 2], with United States of America volumes growing roughly threefold between the mid‐1990s and 2006 [3] and same‐day knee and hip arthroplasty rising from approximately 1%–2% to 54%–62% between 2016 and 2020 [4, 5]. This transition has been driven by the need to contain costs, optimize resources, and improve patient convenience, facilitated by less invasive surgical techniques and safer anesthesia practices [6]. Within this transformed setting, patients consistently rank avoidance of postoperative nausea and vomiting (PONV) and pain among their highest priorities [7, 8], assign substantial monetary value to a symptom‐free recovery [9, 10], and continue to report a measurable gap between expectation and postdischarge experience [11]. For outpatient surgery, selecting an anesthetic regimen that ensures intraoperative stability and high‐quality postoperative recovery is therefore essential, balancing clinical outcomes with practical considerations such as drug costs, operating room turnover, and postanesthesia care unit (PACU) utilization.

The ideal anesthetic agent should provide rapid onset, fast offset with complete recovery of psychomotor functions, minimal AEs, and enable an early and safe discharge. Propofol‐based total intravenous anesthesia (TIVA), often administered via target controlled infusion (TCI), is favored for its smooth recovery profile and its intrinsic antiemetic properties [12]. Conversely, sevoflurane remains the primary volatile halogenated agent of choice due to its low solubility and favorable pharmacokinetics compared to older inhalational anesthetics [13]. While desflurane exhibits a theoretically faster recovery profile [14], its higher costs, significant global warming potential, and the regulatory restrictions in Europe (effective from January 2026) have established sevoflurane as the primary volatile comparator [15].

Despite the widespread use of both techniques, significant controversy persists regarding their relative impact on recovery. PONV is one of the most common and distressing complications following general anesthesia, remaining a major barrier to same‐day discharge [16], with a lower incidence in propofol‐based TIVA [17]. While some evidence suggests that TIVA may provide superior early postoperative quality of recovery in inpatient surgery [18, 19], other investigations have demonstrated comparable recovery profiles, particularly when appropriate depth‐of‐anesthesia monitoring is used [20, 21]. Despite the substantial body of literature comparing propofol and sevoflurane in ambulatory surgery, significant heterogeneity exists in study designs, patient populations, outcome measures, and reported findings, hindering the formulation of definitive evidence‐based practice recommendations [6, 18, 22, 23]. Although a systematic review on this topic was already published in 2014 showing lower PONV incidence and shorter hospital stay with TIVA, several randomized controlled trials (RCTs) have been published afterward.

Given these conflicting results and new published evidence, there is a clear need for a contemporary synthesis of evidence to guide clinical practice, and contemporary comparison of TIVA and sevoflurane in day‐case surgery must also be evaluated against patient‐centered priorities. This systematic review and meta‐analysis aims to comprehensively compare propofol‐based TIVA with sevoflurane‐based inhalational anesthesia in ambulatory surgery, by evaluating recovery kinetics, AEs, and quality of recovery. By providing a rigorous qualitative and quantitative analyses of the available literature, this study intends to offer clinicians a comprehensive, evidence‐based guide for optimal anesthetic selection in the ambulatory surgical setting, with the ultimate goal of improving patient outcomes and healthcare efficiency.

## 2. Materials and Methods

### 2.1. Protocol and Eligibility Criteria

We conducted a systematic review and meta‐analysis in accordance with the Preferred Reporting Items for Systematic Reviews and Meta‐Analyses (PRISMA) guidelines [24]. The PRISMA checklist is included in Supporting Table S1. This systematic review and meta‐analysis followed a preregistered protocol (PROSPERO: CRD420251151311), which predefined the search strategy, inclusion criteria, outcome measures, and analytical plan. The PROSPERO protocol was updated on March 3, 2026 to align the description of the selection process, data extraction, risk of bias assessment, additional outcomes, and planned synthesis with the conduct of the review. The amendment aligned the protocol text with conduct of the meta‐analysis (study selection, data extraction, risk of bias, outcome list, planned synthesis); primary outcomes and the main analytical strategy were not changed from the original registration. Eligibility was restricted to RCTs published in peer‐reviewed journals, in English or Croatian language. The study population comprised adult patients (≥ 18 years) undergoing ambulatory or outpatient surgical procedures. The primary intervention of interest was propofol‐based TIVA, administered via TCI or standard continuous infusion, compared directly against sevoflurane inhalation anesthesia.

Primary and secondary outcomes were prespecified in the PROSPERO protocol (CRD420251151311) and are reported accordingly. Main (primary) outcomes included readiness for discharge (utilizing scales such as PADSS or Aldrete) [25, 26], PACU length of stay (LOS), and emergence time, that was defined by eye opening, obeying commands, or extubation. Additional (secondary) outcomes included the incidence of PONV, postoperative pain scores, rescue antiemetic and analgesic requirements, unplanned hospital admissions, AEs such as hemodynamic or respiratory instability, and all‐cause mortality at the longest follow‐up available. We excluded studies involving inpatient or major surgeries, studies with non‐RCT designs, and trials utilizing desflurane or other volatile agents. Studies that met the eligibility criteria were further classified into 11 core studies for the primary analysis and four sensitivity studies based on prespecified methodological considerations. Studies were designated as sensitivity studies if they exhibited one or more of the following characteristics that could introduce systematic differences from the main body of evidence: (a) use of a concomitant regional anesthetic block in both treatment arms, confounding the isolated comparison of maintenance anesthetic agents; (b) a primary research question substantially different from the comparison of TIVA versus sevoflurane on recovery outcomes; or (c) primary outcome measures that did not map onto the predefined clinical recovery endpoints of this review; or (d) a crossover randomized design, in which the same participants receive both anesthetic techniques in separate periods, precluding pooling with parallel‐group RCTs in the primary meta‐analyses.

### 2.2. Information Sources and Search Strategy

A comprehensive literature search was conducted through PubMed, Embase, the Cochrane Central Register of Controlled Trials (CENTRAL), Scopus, and Web of Science (WOS). Additionally, trial registries including ClinicalTrials.gov and the WHO International Clinical Trials Registry Platform (ICTRP) were consulted. The search strategy integrated Medical Subject Headings (MeSH) and free‐text keywords related to propofol, TIVA, sevoflurane, ambulatory surgery, and recovery outcomes. Search terms included “propofol,” “total intravenous anesthesia,” “TIVA,” “sevoflurane,” “ambulatory surgery,” “day case surgery,” “outpatient surgery,” “recovery,” “postoperative nausea,” “discharge readiness,” and related terms. As prespecified in the PROSPERO protocol, the search was limited to studies published from the January 1, 2014 until the September 19, 2025. This date restriction was chosen because a comprehensive meta‐analysis on this topic was last published in 2014 [6], and we aimed to focus on evidence published since that time to evaluate contemporary anesthetic practices and ensure relevance to current clinical practice.

### 2.3. Study Selection and Data Extraction

The selection process followed a rigorous two‐stage process. In the first stage (title and abstract screening), two independent and blinded reviewers screened all retrieved articles based on titles and abstracts, achieving substantial inter‐rater agreement (*κ* = 0.82, 95% CI: 0.76–0.88). In the second stage, potentially eligible studies identified in the first stage were retrieved in full text and assessed for eligibility by two independent and blinded reviewers using prespecified criteria. Inter‐rater agreement for full‐text assessment was substantial (*κ* = 0.78, 95% CI: 0.69–0.87) also in this phase. In both phases, any disagreements were resolved through consensus or consultation with a third expert reviewer. In case of exclusion, the reason was documented.

Data extraction from the final included studies was independently performed by two reviewers using a standardized data extraction form. The following information was extracted: (1) study characteristics (first author, publication year, country, study design, sample size); (2) patient characteristics (age, gender, type of surgical procedure, ASA physical status); (3) intervention details (propofol dosing regimen, depth of anesthesia monitoring, concurrent medications); and (4) comparison details (sevoflurane concentration, propofol target concentration). In case of continuous outcomes, mean, standard deviation (SD), and sample size were recorded; in case of binary outcomes, number of events and total participants for each group were recorded; in case of time‐to‐event outcomes, event rates and follow‐up duration were recorded. Methodological details regarding randomization, concealment, and blinding were also recorded.

### 2.4. Risk of Bias Assessment

The methodological quality of the included RCTs was evaluated using the Cochrane Risk of Bias 2 (RoB 2) tool for RCTs [27]. This assessment covered five key domains: (1) the randomization process, (2) deviations from intended interventions, (3) missing outcome data, (4) measurement of the outcome, (5) and selection of the reported result. Each domain was categorized as “low risk,” “high risk,” or “some concerns.” Reviewers maintained substantial agreement during this process (*κ* = 0.81, 95% CI: 0.74–0.88); disagreements were resolved through discussion. Notably, given the clinical nature of anesthesia, a lack of blinding was not used as a strict criterion for exclusion.

### 2.5. Statistical Analysis

Quantitative synthesis was performed using R statistical software (version 4.5.1) with the “meta” (version 8.2–1) and “metafor” packages (version 4.0–0). Analyses were run by the authors in RStudio. AI‐assisted tools (Claude Opus, by Anthropic) were utilized to polish the wording of the revised manuscript; the authors reviewed the text and take responsibility for it.

Calculations were performed from documented scripts applied to study‐level means, SDs, and event counts extracted from the included trials. Random‐effects models used the meta package (metacont and metabin functions) with REML estimation of tau^2^ and Hartung–Knapp–Sidik–Jonkman (HKSG) 95% confidence intervals (CIs); ratio‐of‐means analyses used the ratio‐of‐means option. Before finalizing the manuscript, the full analysis pipeline was rerun and all pooled estimates in the text, tables, and figures were checked against the script output.

Continuous outcomes (discharge readiness, PACU LOS, emergence time, pain scores) were expressed as mean differences (MD) or standardized mean differences (SMD) both with 95% CI, while binary outcomes (PONV incidence, rescue antiemetics) were reported as risk ratios (RR) with 95% CI.

We employed random‐effects models using the restricted maximum‐likelihood (REML) estimator for tau^2^ to account for anticipated clinical and methodological heterogeneity. Random‐effects 95% CI and *p*‐values used the HKSJ method; prediction intervals were reported for pooled estimates.

Heterogeneity was quantified using the I^2^ statistic—interpreted as low (< 25%), moderate (25%–50%), substantial (50%–75%), or considerable (> 75%)—and Cochran’s Q statistic (with *p* < 0.10 indicating statistical significance) [28, 29]. I^2^ is reported descriptively in light of clinical and methodological diversity and was not interpreted in isolation. To ensure the robustness of our findings, we conducted three sensitivity analyses: (1) leave‐one‐out analysis (meta‐analysis was repeated by systematically excluding each study to assess the influence of individual studies); (2) influence analyses (studies were ranked by their influence on the pooled estimate); and (3) core vs. all studies (comparison of results using only core studies versus all available studies).

Subgroup analyses were performed based on TIVA administration mode (continuous infusion versus TCI) for the primary PONV outcome.

Publication bias was assessed through visual inspection of contour‐enhanced funnel plots; Egger’s and Peters’ tests were applied where at least 10 studies contributed to an outcome [30, 31]. Where necessary, the trim‐and‐fill method was applied to adjust for potentially missing studies [32]. Finally, a cumulative meta‐analysis was performed chronologically. The overall quality of evidence for each outcome was graded according to the GRADE approach [33, 34]. Time‐to‐event recovery outcomes (PACU LOS and emergence) were additionally synthesized as ratios of means on the logarithmic scale, reflecting the approximately log‐normal distribution of these times [35–37]. Where means and SDs were available, ratios of SDs were explored descriptively [36, 37]. A random‐effects meta‐analysis was not performed for outcomes with only one eligible study; such results are reported as individual‐study estimates. The timing and content of PROSPERO amendments are listed in the Supporting appendix. A further amendment on August 11, 2026 (Version 3.0) added ratio‐of‐means analyses for log‐normally distributed recovery times and specified that outcomes informed by a single study would not be analyzed with REML. The 2014 search boundary focuses on contemporary practice after the prior systematic review and is acknowledged as a limitation regarding earlier trials. All tests were two‐tailed with statistical significance set at *p* < 0.05, and CIs were reported at the 95% level.

## 3. Results

### 3.1. Study Selection and Characteristics

The initial literature search yielded a total of 582 records, of which 15 studies published between 2014 and 2025 met all eligibility criteria and were included in the final meta‐analysis (Table 1) [23, 38–51]. The study selection process is represented in the PRISMA flow diagram (Figure 1). The excluded studies are presented in Table S2.

**TABLE 1:** Characteristics of the 11 studies included in the primary analysis.

Study	Type of study	Country	N. patients total	N. TIVA	N. Sevoflurane	Surgery type	Setting	ASA	TIVA mode	Antiemetic prophylaxis
Castillo PJ 2014	RCT	Spain	59	29	30	Inguinal hernia surgery	Day Surgery	NR	TCI	None
Shen L 2014	RCT	China	120	60	60	Laparoscopic gynecology	Day Surgery	1‐2	TCI	None
Çaparlar CÖ 2016	RCT	Turkey	79	39	40	Laparoscopic cholecystectomy	Day Surgery	1‐3	Manual	Metoclopramide
De Olivera GS 2017	RCT	USA	67	37	30	Gynecological surgery	Day Surgery	1‐2	Manual	Dexamethasone, ondansetron
Chung PK 2018	RCT	Australia	100	50	50	Dental surgery	Day Surgery	1‐2	TCI	Dexamethasone, ondansetron
Shah J 2018	RCT	India	50	25	25	Laparoscopic cholecystectomy	Day Surgery	1‐2	Manual	None
Besnier E 2020	RCT	France	116	58	58	Gynecological surgery	Day Surgery	1‐2	TCI	Dexamethasone, droperidol
Kim H 2020	RCT	Korea	99	50	49	Laparoscopic gynecology	Day Surgery	1‐2	Manual	Dexamethasone
Mustapure N 2024	RCT	India	50	25	25	Minor Surgery	Day Surgery	1‐2	Manual	None
Kumar G 2025	RCT	India	110	55	55	Laparoscopic surgery	Day Surgery	1‐2	Manual	None
Lakshmi KV 2025	RCT	India	64	32	32	Laparoscopic surgery	Day Surgery	1‐2	Manual	None

Abbreviation: RCT, randomized controlled trial.

**FIGURE 1:**
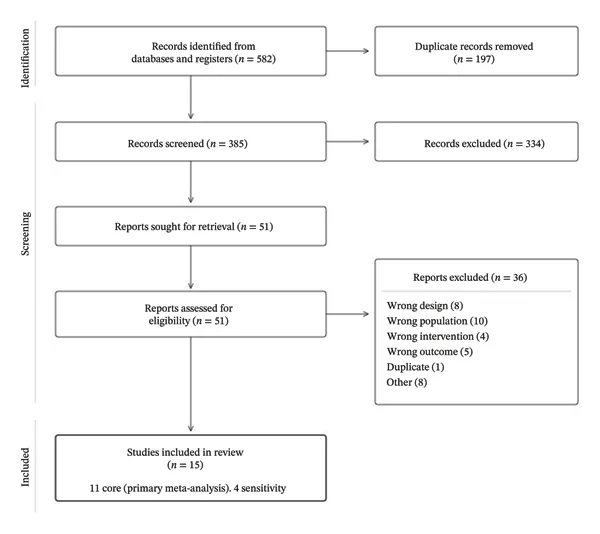
PRISMA 2020 flow diagram showing the study selection process. Reasons for full‐text exclusion (*n* = 36): wrong design (8); wrong population (10); wrong intervention (4); wrong outcome (5); duplicate (1); other (8). See Supporting Table S4. PROSPERO CRD420251151311.

The 15 studies included in the meta‐analysis involved patients randomized to either propofol‐based TIVA or sevoflurane across various ambulatory surgical specialties, including general, orthopedic, gynecological, and urological procedures. The studies were further categorized into (1) 11 core studies for primary analyses [23, 38–40, 42–48] and (2) four studies reserved for sensitivity assessments to ensure the robustness of the findings, based on the prespecified criteria outlined in Section 2.1 [41, 49–51].

Methodological quality was evaluated using the Cochrane ROB 2 tool (Figure 2). A GRADE summary of findings table presents certainty ratings and effect estimates using HKSJ intervals (Table 2).

**FIGURE 2:**
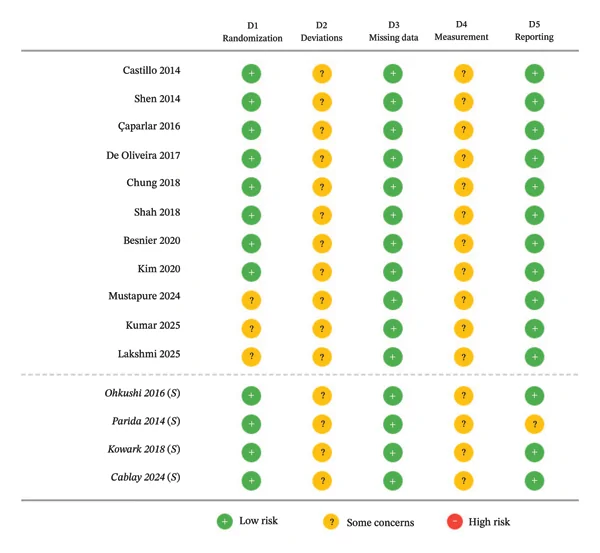
Risk of bias summary (RoB 2) for included randomized trials. (S) = sensitivity analysis only. D2 and D4 rated “some concerns” across all trials due to infeasibility of blinding the anesthetic technique.

**TABLE 2:** GRADE summary of findings.

Outcome	Effect estimate (HKSJ 95% CI)	*p* value	Participants (studies)	Certainty (GRADE)	Main footnote
Readiness for discharge (min)^∗^	MD ‐5.80 (−10.63 to −0.97); ROM 0.925 (0.866–0.987)	0.019	99 (1)	Low	Single‐study finding [45]; not a pooled random‐effects estimate.

PACU length of stay (min)	MD ‐1.26 (−7.34 to 4.83); ROM 0.92 (0.70–1.21)	0.56/0.41	323 (4)	Low	High heterogeneity (I^2^ MD 83.3%; I^2^ ROM 88.4%); CI crosses the null.

Emergence time (min)	MD 1.06 (−2.16–4.28); ROM 1.11 (0.75–1.64)	0.46/0.56	652 (8)	Very low	Extreme heterogeneity (I^2^ ≈ 98.5%); not intended for pooled clinical inference.

PONV incidence	RR 0.67 (0.47–0.96); RD ‐0.107 (−0.177 to −0.036); NNT ≈ 9.4; trim‐fill adjusted RR 0.78 (0.55–1.10)	0.034/0.138	791 (9)	Low	Small‐study effects present; trim‐and‐fill CI includes 1.

Rescue antiemetics	RR 0.55 (0.19–1.62)	0.18	445 (4)	Low	Imprecision; CI crosses the null. Estimate derived from core HKSJ analysis.

Postoperative pain	SMD ‐0.30 (−0.51 to −0.09); VAS 0–10 MD ‐0.29 (−0.50 to −0.07)	0.017/0.023	423 (5 SMD; 4 VAS)	Moderate	Highly consistent SMD (I^2^ = 0); absolute reduction is modest (∼0.3 points on a 0‐10 VAS).

*Note:* Artung–Knapp–Sidik–Jonkman intervals are presented where applicable. *p*‐values are presented for main pooled analyses/secondary parameter adjustments (e.g., ratio of means or trim‐and‐fill model) where applicable. Single *p*‐values represent direct single‐study estimates (e.g., readiness for discharge), whereas dual *p*‐values reflect the primary pooled model alongside sensitivity/adjustment models (such as ratio of means or trim‐and‐fill adjustments). HKSJ, Hartung–Knapp–Sidik–Jonkman adjustment.

Abbreviations: CI, confidence interval; GRADE, Grading of Recommendations Assessment, Development and Evaluation; MD, mean difference; NNT, number needed to treat; PACU, postanesthesia care unit; PONV, postoperative nausea and vomiting; RD, risk difference; ROM, ratio of means; RR, risk ratio; SMD, standardized mean difference; VAS, visual analog scale.

^∗^Discharge readiness is based on a single core trial [45] and does not represent a pooled meta‐analytic estimate.

### 3.2. Primary Outcomes

#### 3.2.1. Readiness for Discharge

Only one core study (Kim et al., *n* = 99: 50 TIVA, 49 sevoflurane) reported extractable means and SDs for discharge readiness. This is an individual‐study result, not a pooled random‐effects meta‐analysis: MD −5.80 min (95% CI −10.63 to −0.97; *p* = 0.019); ratio of means 0.93 (95% CI 0.87–0.99).

#### 3.2.2. PACU LOS

In contrast, PACU LOS, reported by four core studies (*n* = 323 patients: 162 TIVA, 161 sevoflurane), did not differ significantly between groups (MD −1.26 min; 95% CI −7.34 to 4.83; HKSJ; *p* = 0.56). This analysis was characterized by substantial heterogeneity with I^2^ = 83.3% (95% CI 57.5%–93.4%), tau^2^ = 10.75, Q = 17.97, *p* = 0.0004 (Figure S1). On the ratio‐of‐means scale, the pooled estimate was 0.92 (95% CI 0.70–1.21; I^2^ = 88%); substantial heterogeneity persisted after log‐scale analysis (Supporting Figure S13).

#### 3.2.3. Emergence Time

Eight studies (*n* = 652) reported data on emergence time. Due to extreme between‐study heterogeneity I^2^ = 98.5%, a pooled MD was not considered appropriate for primary clinical interpretation. An exploratory analysis using ratios of means yielded a pooled ratio of 1.11 (95% CI 0.75–1.64; *k* = 8), yet heterogeneity remained extreme (I^2^ ≈ 98%), reinforcing that no clinically meaningful pooled inference can be drawn. Individual study‐level MDs (TIVA minus sevoflurane) ranged from −2.9 to 8.0 min, reflecting significant inconsistency in emergence kinetics across the included trials, primarily driven by outcome‐definition heterogeneity (Figure S2; Figure S14).

### 3.3. Secondary Outcomes

#### 3.3.1. PONV Incidence

Nine core studies (*n* = 791: 399 TIVA, 392 sevoflurane) reported PONV incidence. Pooled incidence was lower with TIVA (RR 0.67; 95% CI 0.47–0.96; HKSJ; *p* = 0.034), corresponding to a relative reduction of about 33% that should be interpreted with caution given small‐study effects (Figure 3).

**FIGURE 3:**
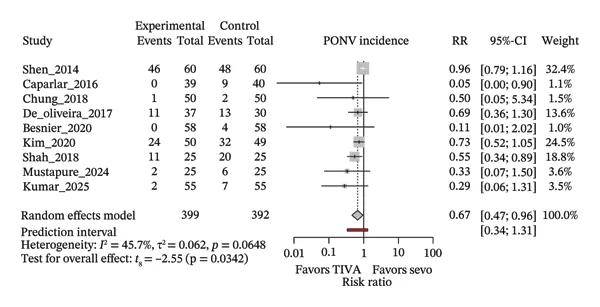
Forest plot: PONV incidence.

Publication bias for the PONV outcome was assessed. Visual inspection of the funnel plot suggested some asymmetry (Figure 4; Figure S3). With *k* = 9, Egger’s and Peters’ tests were not reported for the primary core pool. Trim‐and‐fill imputed five studies (adjusted RR 0.78; 95% CI 0.55–1.10; *p* = 0.138) (Figure S4).

**FIGURE 4:**
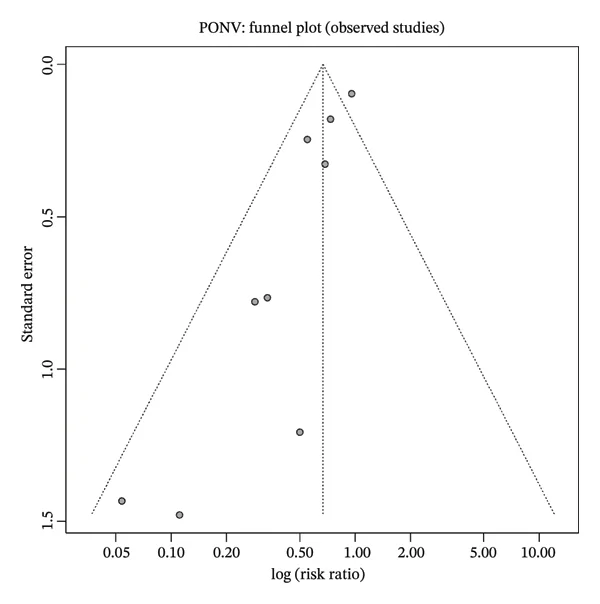
Funnel plot: PONV incidence.

#### 3.3.2. Rescue Antiemetic Administration

Four core studies (*n* = 445 participants) reported rescue antiemetic administration. While there was a downward trend in the requirement for rescue antiemetics with TIVA (RR 0.55; 95% CI 0.19–1.63; HKSJ), this did not reach statistical significance (*p* = 0.18) (Figure S5).

#### 3.3.3. Postoperative Pain Scores

Postoperative pain management also favored the TIVA approach. Based on five core studies (*n* = 423), propofol was associated with significantly lower pain scores (SMD −0.30; 95% CI −0.51 to −0.09; HKSJ; *p* = 0.017). Heterogeneity was absent (I^2^ = 0%) for this outcome. The corresponding absolute difference among VAS 0–10 trials was small (MD −0.29 points; 95% CI −0.50 to −0.07) (Figure 5).

**FIGURE 5:**
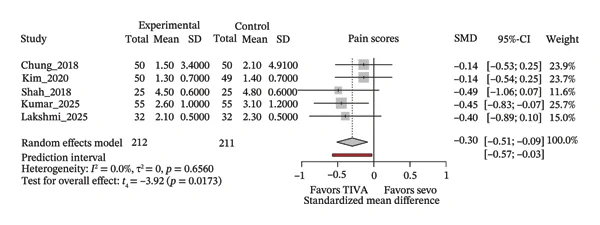
Forest plot: pain scores.

#### 3.3.4. Sensitivity Analyses

Data regarding sensitivity analyses performed in this meta‐analysis are presented in Figures S6–S12.

#### 3.3.5. Mortality

None of the 15 included studies reported mortality as a primary or secondary outcome. It is noteworthy that there was a complete lack of data regarding long‐term follow‐up or survival, suggesting that trialists may have omitted these safety metrics from their reporting or failed to perform adequate postoperative monitoring beyond the immediate discharge period.

### 3.4. Summary

In summary, this systematic review and meta‐analysis of 15 studies suggests that propofol TIVA may improve selected recovery‐related symptomatic outcomes in ambulatory surgery compared to sevoflurane.

The primary findings show a lower pooled PONV incidence (RR 0.67; 95% CI 0.47–0.96; relative reduction 33%, attenuated under trim‐and‐fill to RR 0.78; 95% CI 0.55–1.10) and also in postoperative pain scores (SMD −0.30; 95% CI −0.51 to −0.09) with propofol‐based TIVA compared to sevoflurane. For discharge readiness, one core study favored TIVA (MD ‐5.80 min; 95% CI −10.63 to −0.97). PACU LOS and emergence did not show consistent clinically important differences. Table 3 summarizes the results of this meta‐analysis.

**TABLE 3:** Summary of pooled meta‐analysis estimates for primary and secondary outcomes.

Outcome	Measure	*k*	Estimate (95% CI)	*p* value	I^2^	Comment
Discharge readiness	MD (min)	1	−5.80 (−10.63 to −0.97)	0.019	—	Single core trial [45]; not pooled
Discharge readiness	ROM	1	0.92 (0.87–0.99)	0.019	—	Single core trial [45]; not pooled
PACU length of stay	MD (min)	4	−1.26 (−7.34 to 4.83)	0.56	83%	Core analysis
PACU length of stay	ROM	4	0.92 (0.70–1.21)	0.41	88%	Supporting Figure S13
Emergence time	MD (min)	8	1.06 (−2.16–4.28)	0.46	98.5%	Extreme heterogeneity; not for pooled inference
Emergence time	ROM	8	1.11 (0.75–1.64)	0.56	98%	Supporting Figure S14
PONV incidence	RR	9	0.67 (0.47–0.96)	0.034	46%	Figure 3
PONV incidence	RD	9	−0.107 (−0.177 to −0.036)	0.008	46%	Control event rate 36%; TIVA 24%. NNT ≈ 9.4
PONV incidence	RR, trim‐and‐fill	14	0.78 (0.55–1.10)	0.138	46%	Sensitivity analysis; interval spans the null
Pain scores	SMD	5	−0.30 (−0.51 to −0.09)	0.017	0%	Figure 5
Pain scores	MD (points)	4	−0.29 (−0.50 to −0.07)	0.023	0%	Absolute scale (0–10 VAS)

*Note:* Hartung–Knapp–Sidik–Jonkman (HKSJ) adjustment applied to random‐effects models where applicable. Discharge readiness values represent single‐study estimates [45] rather than a pooled meta‐analytic effect. *k*: number of included studies; PONV: postoperative nausea and vomiting.

Abbreviations: CI, confidence interval; MD, mean difference; PACU, postanesthesia care unit; RD, risk difference; ROM, ratio of means; RR, risk ratio; SMD, standardized mean difference; VAS, visual analog scale.

## 4. Discussion

### 4.1. Summary of Main Findings

This meta‐analysis pooled data from 15 RCTs comparing propofol TIVA and sevoflurane in outpatient surgery.

A reduction in PONV was the most consistent finding; in particular, PONV incidence in propofol‐based TIVA was reduced by about 33% in nine core studies with 791 participants (RR 0.67), but trim‐and‐fill moved the estimate toward the null (adjusted RR 0.78; 95% CI 0.55–1.10). We also noted a trend toward the reduced use of rescue antiemetics with TIVA, although not statistically significant meaning that we cannot draw firm conclusions about this effect.

Propofol TIVA was associated with lower postoperative pain scores with a small effect size that was consistent across studies; certainty is limited by a lack of blinding of anesthesia providers and related performance/detection concerns, with GRADE ratings shown in Table 2. Despite statistical robustness, the modest effect size means that we should interpret this with caution for clinical practice.

Recovery outcomes were mixed. Discharge readiness was shorter with TIVA in the single contributing core trial [45], meaning this finding represents a single‐study result rather than a pooled estimate. PACU LOS did not show significant differences. Emergence time was not pooled owing to extreme heterogeneity.

### 4.2. Interpretation in the Context of Existing Evidence

The lower rates of PONV with propofol TIVA are consistent with the previous results of other studies regarding the well‐known antiemetic effects of propofol [6]. Similar reductions in PONV occur when propofol replaces volatile agents in both inpatient and outpatient settings [52–54]. The 33% risk reduction we found is clinically important because PONV often delays discharge and causes dissatisfaction in ambulatory surgery [16], but the estimate is sensitive to small‐study effects.

The finding of pain reduction is recent and requires careful interpretation. The effect is modest, but the lack of variability across studies must be weighed against unblinded providers and modest absolute differences. We cannot determine the mechanism from our data, although it could be related to the effects of propofol on inflammation, pain pathways, or opioid use. Some laboratory studies suggest that propofol can reduce the production of proinflammatory cytokines by inhibiting the NF‐κB pathway [55], but we do not know if this translates to a significant reduction in pain in patients. More research is needed to understand its clinical relevance.

Time to awakening and PACU LOS were not significantly different between propofol TIVA and sevoflurane, consistent with the pharmacokinetic profiles of both agents. The low blood–gas solubility coefficient of sevoflurane (0.65) allows for relatively rapid elimination, which may minimize differences in early recovery compared with propofol [13]. The extremely high heterogeneity for time to awakening (I^2^ = 98.5%) indicates considerable variation in how studies have measured this outcome. Definitions varied across studies; indeed, some used eye opening, others response to commands, and some time to extubation. Assessment procedures and patient factors also contributed. This level of heterogeneity means that our pooled estimates are not interpretable and suggests that time to awakening depends on factors beyond the choice of maintenance anesthetic.

The point estimate from one core study is compatible with faster fulfillment of discharge criteria when PONV is reduced, with a statistically significant interval in that single‐study pool. Causal interpretation is therefore limited. The lack of a significant difference in PACU LOS, despite the statistically significant reduction in time to discharge readiness, suggests that the actual duration of recovery is often dictated by logistical constraints rather than clinical recovery alone. Factors such as bed availability in recovery areas, transport coordination, and institutional discharge protocols may delay physical exit from the PACU even after patients have met formal discharge criteria.

In the latest meta‐analysis on the same topic, Kumar and colleagues found a 44% reduction in PONV, as well as a shorter time of total hospital stay and time to discharge (MD −14 min), although no statistically significant difference was found in postoperative pain scores, when comparing TIVA to sevoflurane. They also reported a higher cost for TIVA (MD €8.16) [6].

### 4.3. Clinical Relevance and Practical Implications

These results are particularly important in outpatient surgery, where rapid recovery, early discharge, and patient satisfaction are priorities. A lower PONV risk with propofol TIVA would matter in ambulatory care, since PONV is common, distressing, and can delay discharge [56]. The relative reduction of about 33% in the core analysis should be read with the trim‐and‐fill result, which moved the estimate toward the null.

Reducing PONV may provide practical benefits: reduced need for rescue antiemetics, faster achievement of discharge criteria, improved patient satisfaction, and potentially lower healthcare costs. These benefits, however, need to be balanced against the logistical challenges of TIVA, including the need for specialized infusion pumps and the steeper learning curve with TCI systems [57]. In practice, limited access to TCI technology in some institutions may constrain the broad implementation of these findings.

Since discharge readiness in the primary analysis rests on only one well‐sized core trial, generalization is limited. Similarly, the observed reduction in postoperative pain, although statistically significant, may carry limited clinical relevance given the modest effect size (SMD ‐0.30). It is well established that for numeric rating scale pain, minimal clinically important differences (MCIDs) are context‐dependent and vary significantly across surgical settings [58]. Since our pooled SMD is modest in standardized units, its clinical importance should be judged cautiously alongside absolute pain scores, as such a difference may not necessarily translate into a perceptible improvement in patient comfort during the immediate postoperative period. Taken together, the data on PACU stay and emergence are limited and inconsistent rather than evidence of equivalence. We therefore separate postoperative comfort (PONV and pain) from recovery kinetics (discharge readiness, PACU LOS, emergence). Consequently, while statistical differences exist, they may not necessarily represent a clear clinical advantage for one technique over the other in terms of recovery speed.

It should also be noted that secondary outcomes such as PONV and pain are highly influenced by baseline patient risk, the type of surgical stimulus, and the heterogeneity of perioperative analgesic and antiemetic protocols across the included studies, which may further attenuate the clinical weight of these findings.

PONV and pain are important patient‐centered outcomes in ambulatory surgery. This review addresses those symptomatic endpoints and cannot evaluate rare major safety outcomes. None of the included ambulatory trials reported mortality, which is expected to be rare in this setting.

Although mortality was not reported in the included ambulatory trials, external meta‐analytical evidence from broader surgical populations suggests that propofol‐based anesthesia might be associated with a relative increase in mortality of approximately 10% compared to volatile anesthetics [59], potentially driven by mitochondrial dysfunction and systemic inflammatory modulation [60]. While these findings cannot be directly extrapolated to low‐risk ambulatory settings, they underscore the need for rigorous long‐term safety monitoring in future trials.

In clinical practice, the choice of anesthetic drugs should be tailored to patient factors, risk of PONV, institutional resources, and cost. Consequently, while propofol TIVA offers clear advantages for patients at a high risk of PONV, these short‐term symptomatic benefits should be weighed against overall safety profiles and broader clinical outcomes. In low‐risk populations or patients with complex comorbidities, volatile anesthesia remains a valid choice, highlighting the need for an individualized approach rather than a one‐size‐fits‐all anesthetic strategy.

### 4.4. Heterogeneity and Methodological Considerations

The very high heterogeneity in time to awakening and in PACU LOS mean that we should interpret these results with caution. For time to awakening, this likely reflects differences in outcome definitions, measurement protocols, and patient populations. Our subgroup and meta‐regression analyses did not identify significant sources of heterogeneity, suggesting that the variation may come from unmeasured factors such as the type of surgical procedure or institutional protocols. We cannot pinpoint specific drivers of this variation from our data.

The moderate heterogeneity for PONV is acceptable and likely reflects differences in baseline risk of PONV, surgical procedures, and antiemetic protocols. Sensitivity analyses confirmed the robustness of the results for PONV: no single study drove the outcome. The largest study (Shen 2014) contributed to the heterogeneity but not to the overall effect estimate [39].

Our subgroup analysis comparing the methods of TIVA (TCI versus manual infusion) showed no significant differences, suggesting that the method of propofol administration may not significantly influence outcomes. However, the small number of studies in each subgroup limits our confidence in this finding.

### 4.5. Limitations

Our review has several limitations. First, the number of studies and participants varied across outcomes; readiness for discharge and rescue antiemetics were assessed in relatively few studies, which may limit the precision and generalizability of our findings for these outcomes.

For PONV, the primary core pool included nine studies; formal Egger’s and Peters’ tests were therefore not emphasized. Funnel asymmetry and trim‐and‐fill (five imputed studies; adjusted RR 0.78; 95% CI 0.55–1.10) indicate that inferences about PONV reduction remain sensitive to small‐study effects. Acknowledging this potential for bias, we downgraded the certainty of evidence for this outcome to “low” according to the GRADE framework (Table 3).

The risk of bias assessment identified “some concerns” across several studies, mainly related to randomization and potential deviations from intended interventions. The inability to blind anesthesiologists to interventions is a known limitation in anesthesia research and can introduce performance bias. Nevertheless, most studies maintained blinding of outcome assessors, which helps reduce detection bias.

We restricted our search to studies published from 2014 onwards, as specified in our PROSPERO protocol, as the most recent comprehensive meta‐analysis on this topic was published in 2014 [6]. While this ensures a focus on recent evidence, it may have excluded earlier relevant studies. We also restricted our search to English and Croatian publications, potentially missing studies in other languages. However, our comprehensive searches of databases and research registries, along with manual reference checking, should have minimized this risk.

Outcome definitions and measurement methods, particularly for time to recovery and readiness for discharge, varied across studies, contributing to heterogeneity. Although this is common in meta‐analyses, this reflects real‐world clinical practice where standard procedures may not always be feasible. Our review focused on outpatient surgery, limiting generalizability to inpatient settings or other populations. Most studies involved short procedures, so our findings may not apply to longer outpatient surgical procedures. Our decision to classify four studies as sensitivity studies, while based on identifiable clinical and methodological differences, introduces a degree of subjectivity in the analytical framework. Although the consistency of findings between the core and sensitivity analyses supports the validity of this approach, we acknowledge that alternative classification schemes could yield different pooled estimates, particularly for outcomes with a small number of contributing studies. Trial sequential analysis was not performed; conventional random‐effects inference does not address required information size.

Finally, none of the included ambulatory trials reported mortality or long‐term follow‐up. Mortality is expected to be rare in this setting, and this review cannot assess it.

### 4.6. Implications for Future Research

Several important questions remain unanswered. Larger, well‐designed RCTs with standardized outcome definitions are needed, particularly for readiness to discharge and time to awakening. These studies should use consistent measurement protocols and include patient‐reported outcomes in addition to objective measures.

The reduction in postoperative pain with propofol TIVA requires further investigation to understand the underlying mechanisms and determine whether this translates into clinically relevant differences in opioid requirements, patient satisfaction, or functional recovery. Cost‐effectiveness analyses comparing propofol TIVA with sevoflurane in ambulatory surgery settings would provide valuable insights, given the potential cost reductions associated with PONV that could offset the higher costs of propofol. Future research should investigate the role of monitoring depth of anesthesia in optimizing outcomes and examine patient satisfaction and quality of recovery using validated instruments, such as QoR‐15 [61] or QoR‐40 [62].

Given the environmental concerns with volatile anesthetics, future research should assess the ecological impact of anesthetic choices, particularly as regulatory restrictions on agents such as desflurane are introduced.

## 5. Conclusions

This systematic review and meta‐analysis of contemporary randomized evidence suggests that propofol‐based TIVA may reduce PONV incidence and is associated with a small reduction in early postoperative pain scores compared with sevoflurane. Absolute PONV reductions correspond to an NNT of about 9 in the core analysis, but small‐study effects and trim‐and‐fill sensitivity analyses urge caution. Discharge readiness was assessed in only one core study and must not be generalized as a pooled advantage of TIVA. PACU LOS did not differ significantly, and emergence times—whether analyzed as MDs or ratios of means—remain too heterogeneous for clinical pooled inference.

Mortality was not reported in the included trials; this review therefore provides no evidence on survival. Findings should also be interpreted in light of possible publication bias, heterogeneity for some outcomes, and small study numbers for certain measures.

In clinical practice, the choice of an anesthetic agent should be tailored to the individual patient’s risk profile, institutional resources, and cost considerations. While propofol TIVA is highly attractive for patients at a high risk of PONV, volatile anesthesia remains a reasonable alternative when PONV risk is low. Future trials should use consistent recovery‐time definitions and report patient‐centered symptomatic outcomes alongside rare safety events when feasible.

## Author Contributions

Conceptualization and design: Ivan Šitum and Slobodan Mihaljević.

Methodology/protocol: Ivan Šitum and Martina Perić Šitum.

Literature search: Ivan Šitum and Martina Perić Šitum.

Deduplication and screening: Ivan Šitum, Ante Erceg, and Martina Perić Šitum.

Data extraction: Roberto Giurazza, Ivan Šitum, and Ante Erceg.

Risk of bias assessment: Ivan Šitum and Ante Erceg.

GRADE/certainty of evidence: Ivan Šitum, and Ante Erceg.

Statistical analysis: Ivan Šitum.

Visualization: Ivan Šitum.

Writing–original draft: Roberto Giurazza, and Ivan Šitum.

Writing–review and editing: Ivan Šitum, Roberto Giurazza, Martina Perić Šitum, Ante Erceg, Slobodan Mihaljević, Francesco Coppolino, Marco Rispoli, and Zoran Karlović.

Project administration: Zoran Karlović.

Supervision: Zoran Karlović and Francesco Coppolino.

## Funding

No funding was received for this manuscript. Open access publishing facilitated by Universita degli Studi della Campania Luigi Vanvitelli, as part of the Wiley ‐ CRUI‐CARE agreement.

## Disclosure

All authors have read and agreed to the published version of the manuscript and can attest to the validity of the work.

## Conflicts of Interest

The authors declare no conflicts of interest.

## Supporting Information

Additional supporting information can be found online in the Supporting Information section.

## Supporting information


**Supporting Information** Search Strings, Table S1. PRISMA 2020 checklist. Table S2. Major exclusion with reason for exclusion. Table S3. Characteristics of the four studies included in the sensitivity assessment. Table S4. Trim‐and‐fill sensitivity analysis for publication bias (PONV incidence). Table S5. Study‐level ratios of means for recovery times (PACU length of stay and emergence time). Table S6. Study‐level ratios of standard deviations (RSD) for continuous recovery time endpoints. Figure S1. Forest plot: PACU length of stay. Figure S2. Forest plot: emergence time. Figure S3. Contour‐enhanced funnel plot: PONV incidence. Figure S4. Trim‐and‐fill funnel plot: PONV incidence. Figure S5. Forest plot: rescue antiemetic administration. Figure S6. Sensitivity analysis: leave‐one‐out analysis for PONV incidence. Figure S7. Influence analysis for PONV incidence. Figure S8. Forest plot: subgroup analysis on PONV incidence by TIVA administration mode. Figure S9. Meta‐regression: effect of publication year on PONV RR. Figure S10. Meta‐regression: effect of sample size on PONV RR. Figure S11. Cumulative meta‐analysis of PONV incidence. Figure S12. PONV incidence in core and sensitivity studies. Figure S13. Forest plot: PACU length of stay, ratio of means. Figure S14. Forest plot: emergence time, ratio of means. Supporting references.

## Data Availability

The data that support the findings of this study are available in the supporting information of this article.
