# Supplementary material for: Recovery Kinetics and Postoperative Outcomes of Propofol‐Based Total Intravenous Anesthesia Versus Sevoflurane in Ambulatory Surgery: A Systematic Review and Meta‐Analysis of Randomized Studies
Source: Anesthesiol Res Pract. 2026 Oct 1;2026:2531080. doi: 10.1155/anrp/2531080 (PMC13628567; doi:10.1155/anrp/2531080)
Supplement: Supplementary file 1 [file ANRP-2026-2531080-s001.zip › Suppl append v.4 meta-analysis TIVA vs sevo for amb surg.docx]

**Supplementary Appendix**

Ivan Šitum, Roberto Giurazza, Martina Perić Šitum, et al. Recovery Kinetics and Postoperative Outcomes of Propofol-Based Total Intravenous Anesthesia Versus Sevoflurane in Ambulatory Surgery: A Systematic Review and Meta-Analysis of Randomized Studies

**Table of contents**

[Search Strings	3](#_Toc238758028)

[Table S1. PRISMA 2020 checklist.	7](#_Toc238758029)

[Table S2. Major exclusion with reason for exclusion.	9](#_Toc238758030)

[Table S3. Characteristics of the four studies included in the sensitivity assessment.	13](#_Toc238758031)

[Table S4. Trim-and-fill sensitivity analysis for publication bias (PONV incidence).	14](#_Toc238758032)

[Table S5. Study-level ratios of means for recovery times (PACU length of stay and emergence time)	15](#_Toc238758033)

[Table S6. Study-level ratios of standard deviations (RSD) for continuous recovery time endpoints	18](#_Toc238758034)

[Figure S1. Forest plot: PACU length of stay.	21](#_Toc238758035)

[Figure S2. Forest plot: emergence time.	22](#_Toc238758036)

[Figure S3. Contour-enhanced funnel plot: PONV incidence.	23](#_Toc238758037)

[Figure S4. Trim-and-fill funnel plot: PONV incidence.	24](#_Toc238758038)

[Figure S5. Forest plot: rescue antiemetics administration.	25](#_Toc238758039)

[Figure S6. Sensitivity analysis: leave-one-out analysis for PONV incidence.	26](#_Toc238758040)

[Figure S7. Influence analysis for PONV incidence.	27](#_Toc238758041)

[Figure S8. Forest plot: subgroup analysis on PONV incidence by TIVA administration mode.	28](#_Toc238758042)

[Figure S9. Meta-regression: effect of publication year on PONV RR.	29](#_Toc238758043)

[Figure S10. Meta-regression: effect of sample size on PONV RR.	30](#_Toc238758044)

[Figure S11. Cumulative meta-analysis of PONV incidence.	31](#_Toc238758045)

[Figure S12. PONV incidence in core and sensitivity studies.	32](#_Toc238758046)

[Figure S13. PACU length of stay, ratio of means.	33](#_Toc238758047)

[Figure S14. Emergence time, ratio of means.	34](#_Toc238758048)

[Supplementary references	35](#_Toc238758049)

# Search Strings

PROSPERO: CRD420251151311 | Search date: September 19–24, 2025

Date filter: from 2014 | Languages: English, Croatian

**1. PubMed/MEDLINE (Sep 19, 2025)**

("ambulatory surgery"[tiab] OR "day case"[tiab] OR "day surgery"[tiab] OR "outpatient"[tiab] OR "same-day"[tiab])

AND (propofol[tiab] OR "total intravenous anesthesia"[tiab] OR TIVA[tiab] OR "target controlled infusion"[tiab] OR TCI[tiab])

AND (sevoflurane[tiab] OR "inhalational anesthesia"[tiab])

AND (randomized[tiab] OR randomised[tiab] OR trial[tiab])

AND ("2014/01/01"[Date - Publication] : "3000"[Date - Publication])

Filters: Publication date from 2014; Language: English, Croatian

**2. Embase (Elsevier) (Sep 24, 2025)**

1. ("ambulatory surgery" or "day case" or "day surgery" or outpatient or "same day").tw.

2. (propofol or "total intravenous anesthesia" or TIVA or "target controlled infusion" or TCI).tw.

3. (sevoflurane or "inhalational anesthesia").tw.

4. (randomized or randomised or trial or "clinical trial" or "controlled trial").tw.

5. 1 and 2 and 3 and 4

6. limit 5 to yr="2014-current"

7. limit 6 to (english or croatian)

(.tw. = title and abstract)

**3. Cochrane CENTRAL (Sep 19, 2025)**

(("ambulatory surgery" OR "day case" OR "day surgery" OR outpatient OR "same day")

AND (propofol OR "total intravenous anesthesia" OR TIVA OR "target controlled infusion" OR TCI)

AND (sevoflurane OR "inhalational anesthesia")):ti,ab,kw

Date filter 2014–present applied via Cochrane interface.

**4. Scopus (Sep 19, 2025)**

TITLE-ABS-KEY (("ambulatory surgery" OR "day case" OR "day surgery" OR outpatient OR "same day")

AND (propofol OR "total intravenous anesthesia" OR TIVA OR "target controlled infusion" OR TCI)

AND (sevoflurane OR "inhalational anesthesia") AND (randomized OR randomised OR trial))

AND (PUBYEAR > 2013)

AND (LIMIT-TO (LANGUAGE, "English") OR LIMIT-TO (LANGUAGE, "Croatian"))

**5. Web of Science Core Collection (Sep 19, 2025)**

("ambulatory surgery" OR "day case" OR "day surgery" OR outpatient OR "same day")

AND (propofol OR "total intravenous anesthesia" OR TIVA OR "target controlled infusion" OR TCI)

AND (sevoflurane OR "inhalational anesthesia")

AND (randomized OR randomised OR trial)

Filters: Timespan 2014–2025; Languages: English, Croatian

**6. Clinical trial registries (search terms, not full syntax)**

ClinicalTrials.gov:

Condition: ambulatory surgery OR outpatient surgery OR day surgery

Intervention: propofol AND sevoflurane

Study type: Interventional | From 01/01/2014

WHO ICTRP:

Condition: ambulatory surgery, outpatient surgery, day surgery

Intervention: propofol, total intravenous anesthesia, TIVA, sevoflurane

From 2014

# Table S1. PRISMA 2020 checklist.

| **Section and Topic** | **Item #** | **Checklist item** | **Location where item is reported** |
| --- | --- | --- | --- |
| **TITLE** | | |  |
| Title | 1 | Identify the report as a systematic review. | Page 1 |
| **ABSTRACT** | | |  |
| Abstract | 2 | See the PRISMA 2020 for Abstracts checklist. | Page 4-5 |
| **INTRODUCTION** | | |  |
| Rationale | 3 | Describe the rationale for the review in the context of existing knowledge. | Pages 6-7 |
| Objectives | 4 | Provide an explicit statement of the objective(s) or question(s) the review addresses. | Pages 7 |
| **METHODS** | | |  |
| Eligibility criteria | 5 | Specify the inclusion and exclusion criteria for the review and how studies were grouped for the syntheses. | Page 8-9 |
| Information sources | 6 | Specify all databases, registers, websites, organisations, reference lists and other sources searched or consulted to identify studies. Specify the date when each source was last searched or consulted. | Page 9 |
| Search strategy | 7 | Present the full search strategies for all databases, registers and websites, including any filters and limits used. | Page 9  Supplementary Appendix |
| Selection process | 8 | Specify the methods used to decide whether a study met the inclusion criteria of the review, including how many reviewers screened each record and each report retrieved, whether they worked independently, and if applicable, details of automation tools used in the process. | Page 9-10 |
| Data collection process | 9 | Specify the methods used to collect data from reports, including how many reviewers collected data from each report, whether they worked independently, any processes for obtaining or confirming data from study investigators, and if applicable, details of automation tools used in the process. | Page 9-10 |
| Data items | 10a | List and define all outcomes for which data were sought. Specify whether all results that were compatible with each outcome domain in each study were sought (e.g. for all measures, time points, analyses), and if not, the methods used to decide which results to collect. | Pages 8-9 |
|  | 10b | List and define all other variables for which data were sought (e.g. participant and intervention characteristics, funding sources). Describe any assumptions made about any missing or unclear information. | Pages 9-10 |
| Study risk of bias assessment | 11 | Specify the methods used to assess risk of bias in the included studies, including details of the tool(s) used, how many reviewers assessed each study and whether they worked independently, and if applicable, details of automation tools used in the process. | Page 10 |
| Effect measures | 12 | Specify for each outcome the effect measure(s) (e.g. risk ratio, mean difference) used in the synthesis or presentation of results. | Pages 10-12 |
| Synthesis methods | 13a | Describe the processes used to decide which studies were eligible for each synthesis (e.g. tabulating the study intervention characteristics and comparing against the planned groups for each synthesis (item #5)). | Pages 8-9; 10-12 |
|  | 13b | Describe any methods required to prepare the data for presentation or synthesis, such as handling of missing summary statistics, or data conversions. | Pages 10-12 |
|  | 13c | Describe any methods used to tabulate or visually display results of individual studies and syntheses. | Pages 10-12 |
|  | 13d | Describe any methods used to synthesize results and provide a rationale for the choice(s). If meta-analysis was performed, describe the model(s), method(s) to identify the presence and extent of statistical heterogeneity, and software package(s) used. | Pages 10-12 |
|  | 13e | Describe any methods used to explore possible causes of heterogeneity among study results (e.g. subgroup analysis, meta-regression). | Pages 10-12 |
|  | 13f | Describe any sensitivity analyses conducted to assess robustness of the synthesized results. | Pages 10-12 |
| Reporting bias assessment | 14 | Describe any methods used to assess risk of bias due to missing results in a synthesis (arising from reporting biases). | Page 10-12 |
| Certainty assessment | 15 | Describe any methods used to assess certainty (or confidence) in the body of evidence for an outcome. | Page 10-12 |
| **RESULTS** | | |  |
| Study selection | 16a | Describe the results of the search and selection process, from the number of records identified in the search to the number of studies included in the review, ideally using a flow diagram. | Page 13; Figure 1 |
|  | 16b | Cite studies that might appear to meet the inclusion criteria, but which were excluded, and explain why they were excluded. | Table S2 |
| Study characteristics | 17 | Cite each included study and present its characteristics. | Page 1; Table 1; Table S3 |
| Risk of bias in studies | 18 | Present assessments of risk of bias for each included study. | Figure 2 |
| Results of individual studies | 19 | For all outcomes, present, for each study: (a) summary statistics for each group (where appropriate) and (b) an effect estimate and its precision (e.g. confidence/credible interval), ideally using structured tables or plots. | Pages 13-16; Table 2; Figure 3, 5; Figure S1, S2, S5, S13, S14 |
| Results of syntheses | 20a | For each synthesis, briefly summarise the characteristics and risk of bias among contributing studies. | Figure 2 |
|  | 20b | Present results of all statistical syntheses conducted. If meta-analysis was done, present for each the summary estimate and its precision (e.g. confidence/credible interval) and measures of statistical heterogeneity. If comparing groups, describe the direction of the effect. | Pages 13-16; Table 2 |
|  | 20c | Present results of all investigations of possible causes of heterogeneity among study results. | Page 15; Figures S8, S9, S10 |
|  | 20d | Present results of all sensitivity analyses conducted to assess the robustness of the synthesized results. | Page 15; Figures S6, S7, S11, S12 |
| Reporting biases | 21 | Present assessments of risk of bias due to missing results (arising from reporting biases) for each synthesis assessed. | Figure 4; Figures S3, S4; Table S4 |
| Certainty of evidence | 22 | Present assessments of certainty (or confidence) in the body of evidence for each outcome assessed. | Table 3 |
| **DISCUSSION** | | |  |
| Discussion | 23a | Provide a general interpretation of the results in the context of other evidence. | Pages 17-19 |
|  | 23b | Discuss any limitations of the evidence included in the review. | Pages 20-22 |
|  | 23c | Discuss any limitations of the review processes used. | Pages 21-22 |
|  | 23d | Discuss implications of the results for practice, policy, and future research. | Pages 22-23 |
| **OTHER INFORMATION** | | |  |
| Registration and protocol | 24a | Provide registration information for the review, including register name and registration number, or state that the review was not registered. | Page 8 |
|  | 24b | Indicate where the review protocol can be accessed, or state that a protocol was not prepared. | Page 8 |
|  | 24c | Describe and explain any amendments to information provided at registration or in the protocol. | Pages 8-9 |
| Support | 25 | Describe sources of financial or non-financial support for the review, and the role of the funders or sponsors in the review. | Page 25 |
| Competing interests | 26 | Declare any competing interests of review authors. | Page 25 |
| Availability of data, code and other materials | 27 | Report which of the following are publicly available and where they can be found: template data collection forms; data extracted from included studies; data used for all analyses; analytic code; any other materials used in the review. | Contact [ivsitum@gmail.com](mailto:ivsitum@gmail.com) |

*From:* Page MJ, McKenzie JE, Bossuyt PM, Boutron I, Hoffmann TC, Mulrow CD, et al. The PRISMA 2020 statement: an updated guideline for reporting systematic reviews. BMJ 2021;372:n71. doi: 10.1136/bmj.n71. This work is licensed under CC BY 4.0. To view a copy of this license, visit <https://creativecommons.org/licenses/by/4.0/>

# Table S2. Major exclusion with reason for exclusion.

Of 51 full-text articles assessed for eligibility, 36 were excluded with reasons as follows:

| **Reason for exclusion** | **n** |
| --- | --- |
| Wrong setting (inpatient/major surgery, cardiac surgery) | 10 |
| Not an RCT (review articles, retrospective studies, protocols) | 8 |
| Wrong comparator (desflurane only, no sevoflurane arm) | 4 |
| Missing relevant outcomes | 5 |
| Duplicate publication | 1 |
| Other (wrong intervention, trial registration only, etc.) | 8 |
| Total | 36 |

**List of excluded studies (with reasons)**

| **#** | **Citation (Author, Year, Title)** | **Reason for exclusion** |
| --- | --- | --- |
| [1] | Jiang J et al. 2023. Volatile Versus Total Intravenous Anesthesia on Postoperative Delirium in Adult Patients Undergoing Cardiac Valve Surgery: A Randomized Clinical Trial. | Wrong setting (cardiac surgery). |
| [2] | Zhang Y et al. 2018. Propofol compared with sevoflurane general anaesthesia is associated with decreased delayed neurocognitive recovery in older adults. | Wrong setting (inpatient, major surgery). |
| [3] | Nieuwenhuijs-Moeke G et al. 2017. Propofol-based anaesthesia versus sevoflurane-based anaesthesia for living donor kidney transplantation: results of the VAPOR-1 randomized controlled trial. | Wrong setting (inpatient). |
| [4] | Lindholm E et al. 2014. Analysis of transthoracic echocardiographic data in major vascular surgery from a prospective randomised trial comparing sevoflurane and fentanyl with propofol and remifentanil anaesthesia. | Wrong setting (major vascular surgery); no relevant outcomes measured. |
| [5] | Wang G et al. 2019. Comparison of the effects of sevoflurane and propofol anesthesia on pulmonary function, MMP-9 and postoperative cognition in patients receiving lung cancer resection. | Wrong setting (inpatient); no relevant outcomes. |
| [6] | Yao J et al. 2024. Propofol total intravenous anesthesia vs. sevoflurane inhalation anesthesia: Effects on post-operative cognitive dysfunction and inflammation in geriatric patients undergoing laparoscopic surgery. | Not randomized. |
| [7] | Cao S et al. 2023. Delirium in older patients given propofol or sevoflurane anaesthesia for major cancer surgery: a multicentre randomised trial. | Wrong setting (major surgery). |
| [8] | Franzen S et al. 2022. Renal function during sevoflurane or total intravenous propofol anaesthesia: a single-centre parallel randomised controlled study. | Wrong setting (inpatient); renal outcomes only (no relevant recovery outcomes). |
| [9] | Goswami U et al. 2015. Comparative evaluation of the effects of propofol and sevoflurane on cognitive function and memory in patients undergoing laparoscopic cholecystectomy: A randomised prospective study. | Missing relevant outcomes (no discharge readiness/PACU LOS/emergence time/PONV with suitable numerics). |
| [10] | Munmany M et al. 2016. The use of inhaled sevoflurane during operative hysteroscopy is associated with increased glycine absorption compared to intravenous propofol for maintenance of anesthesia. | Wrong setting (inpatient); wrong outcome (glycine absorption). |
| [11] | CTRI/2022/05/042869. 2022. Quality of recovery in patients undergoing open pelvic surgery under general anaesthesia using total intravenous anaesthesia or full inhalation anaesthesia. | Trial registration only (no full report). |
| [12] | NCT04793022. 2021. Total Intravenous Anesthesia With Propofol vs. General Anesthesia in Outpatient Shoulder Arthroscopic Surgeries. | Trial registration only (no full report). |
| [13] | Na SH et al. 2018. Patient quality of recovery on the day of surgery after propofol total intravenous anesthesia for vitrectomy: a randomized controlled trial. | Wrong comparator (desflurane). |
| [14] | Oliveros H et al. 2020. Variability of anesthetic depth in total intravenous anesthesia vs balanced anesthesia using entropy indices: a randomized, crossover, controlled clinical trial. | Missing relevant outcomes (entropy/narrative outcomes only). |
| [15] | Jones KA et al. 2015. Intravenous Anesthesia With Bispectral Index Monitoring vs Inhalational Anesthesia for Rhytidoplasty: a Randomized Clinical Trial. | Review/not meeting eligibility (pregled). |
| [16] | Prathep S et al. 2015. Target Controlled Infusion versus Sevoflurane/Desflurane Anesthesia for Laparoscopic Cholecystectomy: comparison Postoperative Nausea/Vomiting and Extubation Time. | Not RCT; no appropriate comparator. |
| [17] | Ding F et al. 2021. Effect of propofol-based total intravenous anaesthesia on postoperative cognitive function and sleep quality in elderly patients. | Wrong setting (inpatient). |
| [18] | Effects of Propofol on Early Recovery of Hunger After Surgery (NCT02272166 / Besnier). 2020. | Duplicate publication (duplicate of Besnier 2020). |
| [19] | Saylan S et al. 2024. Effect of classical laryngeal mask or I-gel use on otolaryngeal system in ambulatory inhalation general anesthesia: A prospective study. | Wrong comparison (I-gel vs LMA, not propofol vs sevoflurane). |
| [20] | Kapoor P et al. 2015. Comparison of the effects of inhalational anesthesia with desflurane and total intravenous anesthesia on cardiac biomarkers after aortic valve replacement. | Wrong comparator (desflurane); wrong setting (cardiac). |
| [21] | Kampmeier T et al. 2021. Cost-Effectiveness of Propofol (Diprivan) Versus Inhalational Anesthetics to Maintain General Anesthesia in Noncardiac Surgery in the United States. | Narrative/cost-effectiveness (not RCT of recovery outcomes). |
| [22] | Piri E et al. 2015. Comparison of Propofol and Desflurane Anesthesia Combined with Remifentanyl in Breast Surgery. | Wrong comparator (desflurane only). |
| [23] | Miller TE et al. 2015. Total Intravenous Anesthesia and Anesthetic Outcomes. | Review article. |
| [24] | Saravanan B et al. 2018. Effect of anaesthetic agents on olfactory threshold and identification - A single blinded randomised controlled study. | Missing relevant outcomes; short procedures not explicit day-case (excluded from quantitative meta-analysis). |
| [25] | NCT04307238. 2019. Effects of Different Types of General Anesthesia on Postoperative Pupillary Reactivity. | Trial registration only (no full report). |
| [26] | Baxter M et al. 2022. Cognitive Recovery by Decade in Healthy 40- to 80-Year-Old Volunteers After Anesthesia Without Surgery. | Wrong intervention (no TIVA comparison as per inclusion). |
| [27] | Lasala J et al. 2020. Impact of anesthesia technique on post-operative opioid use in open gynecologic surgery in an enhanced recovery after surgery pathway. | Retrospective study (not RCT). |
| [28] | Shi Y et al. 2019. Application of different anesthetic methods in coronary artery bypass grafting and the effect on postoperative outcome. | Wrong setting (CABG / cardiac surgery). |
| [29] | Attri J et al. 2017. Application of Discharge Criteria for Home Readiness Using Bispectral and Supraglottic Airway Devices in Day-care Surgery without Using Muscle Relaxants. | Wrong comparator (desflurane). |
| [30] | Adis Medical Writers. 2018. Balance risks and benefits when selecting anaesthetic and perioperative care options in elderly surgical outpatients. Drugs Ther Perspect. | Review article. |
| [31] | No author. 2019. International Anesthesia Research Society 2019 Annual Meeting and International Science Symposium. Anesth Analg. | Abstract book / conference (not full article). |
| [32] | EUCTR2018-001252-35-DK. 2018. Changes in the human brain induced by general anaesthesia. | Review / not full trial report. |
| [33] | NCT03441828. 2018. Effects of Depth of Neuromuscular Block on Surgical Operating Conditions in Gynecologic Laparoscopic Surgery. | Wrong topic (neuromuscular block; not propofol vs sevoflurane). |
| [34] | Vaughan J et al. Anaesthetic regimens for day-procedure laparoscopic cholecystectomy. | Review article. |
| [35] | Shu et al. 2024. Comparison of sevoflurane and propofol in combination with remifentanil on the quality of postoperative recovery in patients undergoing laparoscopic bariatric surgery. | Missing relevant outcomes (no outcome measured as required). |
| [36] | Besir A et al. 2017. Comparison of surgical conditions in 2 different anesthesia techniques of esmolol-induced controlled hypotension in breast reduction surgery. | After verification: wrong comparison (esmolol-induced hypotension / surgical conditions; not propofol vs sevoflurane for recovery outcomes). |

Total: 36 (matches protocol).

Note:

This table was populated from the screening file fulltext_retrieval_wnakon pyton screen - fulltext_retrieval_with_rct_status is.csv (column F = continues after individual full-text screening, 0 = excluded, reason in G; column H = continues after full-text reading, 0 = excluded, reason in I). Reasons are given as in the source; some were translated or standardized for PRISMA (wrong setting, not RCT, wrong comparator, missing outcomes, duplicate, other).

**Exclusion reason categories (use consistently):**

Wrong setting — Inpatient, major surgery, cardiac surgery, or non-ambulatory setting

Not RCT — Review, retrospective study, protocol without results, or non-randomized design

Wrong comparator — Desflurane (or other volatile) only, no sevoflurane arm

Missing outcomes — No relevant recovery/ambulatory outcomes reported

Duplicate — Duplicate publication of an already included study

Other — Wrong intervention (e.g. propofol induction only), trial registration only, or other (specify in table)

# Table S3. Characteristics of the four studies included in the sensitivity assessment.

| Study | Type of study | Country | N. patients total | N. TIVA | N. Sevoflurane | Surgery Type | Setting | ASA risk class | TIVA Mode | Antiemetic Prophylaxis |
| --- | --- | --- | --- | --- | --- | --- | --- | --- | --- | --- |
| Parida S 2014 | RCT | NR | 100 | 50 | 50 | Mixed day-care surgery | Day Surgery | NR | NR | NR |
| Ohkushi K 2016 | Cross-over study | Japan | 20 | 20 | 20 | Dental surgery | Day Surgery | 1-2 | TCI | NR |
| Kowark A 2018 | RCT | Germany | 343 | 118 | 111 (+114 desflurane) | Mixed day-care surgery | Day Surgery | NR | TCI | NR |
| Cablay KJ 2024 | RCT | USA | 82 | 42 | 40 | Shoulder arthroscopy | Day Surgery | NR | TCI | NR |

RCT: randomized controlled trial; NR: not reported; TCI: Target Controlled Infusion; ASA: American Society of Anesthesiology

# Table S4. Trim-and-fill sensitivity analysis for publication bias (PONV incidence).

| Parameter | Value |
| --- | --- |
| Outcome evaluated | PONV incidence |
| k | 9 |
| Imputed studies (theoretical missing trials) | 5 |
| k_adj_ | 14 |
| Adjusted pooled RR | 0.78 |
| 95% CI | 0.55 to 1.10 |
| Adjusted p-value | 0.138 |
| Interpretation | The 95% CI spans the null value (1.0); the adjusted estimate moves toward the null, indicating that small-study effects attenuate the observed antiemetic benefit of TIVA. |

PONV: postoperative nausea and vomiting; k: studies in core analysis; k_adj_: total studies after trim-and-fill; RR: risk ratio; CI: confidence interval; TIVA: total intravenous anesthesia.

# Table S5. Study-level ratios of means for recovery times (PACU length of stay and emergence time)

**Part A: Core Studies**

| Outcome | Study ID | N. (TIVA) | N. (Sevo) | Mean TIVA (min) | Mean Sevo (min) | SD TIVA | SD Sevo | ROM | 95% CI |
| --- | --- | --- | --- | --- | --- | --- | --- | --- | --- |
| PACU length of stay | Kim 2020 | 50 | 49 | 15.7 | 17.0 | 3.0 | 3.6 | 1.32 | 0.85 to 1.00 |
|  | Mustapure 2024 | 25 | 25 | 35.4 | 40.5 | 10.2 | 12.0 | 1.27 | 0.74 to 1.03 |
|  | Kumar 2025 | 55 | 55 | 11.4 | 14.8 | 2.7 | 3.5 | 1.17 | 0.71 to 0.84 |
|  | Lakshmi 2025 | 32 | 32 | 29.5 | 25.2 | 8.0 | 7.5 | 1.17 | 1.02 to 1.35 |
| Emergence time | Shen 2014 | 60 | 60 | 13.07 | 9.20 | 6.56 | 4.40 | 1.38 | 1.15 to 1.65 |
|  | Chung 2018 | 50 | 50 | 2.5 | 2.9 | 1.0 | 0.9 | 1.26 | 0.75 to 0.99 |
|  | Castillo 2014 | 29 | 30 | 17.0 | 9.0 | 7.0 | 3.0 | 2.29 | 1.56 to 2.29 |
|  | Shah 2018 | 25 | 25 | 8.0 | 3.4 | 0.7 | 1.2 | 2.35 | 2.04 to 2.71 |
|  | Mustapure 2024 | 25 | 25 | 8.5 | 11.4 | 2.3 | 2.7 | 1.15 | 0.65 to 0.86 |
|  | Kumar 2025 | 55 | 55 | 6.1 | 8.3 | 1.8 | 2.2 | 1.13 | 0.66 to 0.82 |
|  | Lakshmi 2025 | 32 | 32 | 7.5 | 6.2 | 1.6 | 1.4 | 1.21 | 1.09 to 1.35 |
|  | Kim 2020 | 50 | 49 | 4.9 | 7.4 | 1.3 | 1.3 | 1.06 | 0.61 to 0.72 |

**Part B: Sensitivity Analysis (All Eligible Studies)**

| Outcome | Study ID | N (TIVA) | N (Sevo) | Mean TIVA (min) | Mean Sevo (min) | SD TIVA | SD Sevo | ROM | 95% CI |
| --- | --- | --- | --- | --- | --- | --- | --- | --- | --- |
| PACU length of stay | Kim 2020 | 50 | 49 | 15.7 | 17.0 | 3.0 | 3.6 | 1.32 | 0.85 to 1.00 |
|  | Mustapure 2024 | 25 | 25 | 35.4 | 40.5 | 10.2 | 12.0 | 1.27 | 0.74 to 1.03 |
|  | Kumar 2025 | 55 | 55 | 11.4 | 14.8 | 2.7 | 3.5 | 1.17 | 0.71 to 0.84 |
|  | Lakshmi 2025 | 32 | 32 | 29.5 | 25.2 | 8.0 | 7.5 | 1.17 | 1.02 to 1.35 |
|  | Kowark 2018 [S] | 118 | 111 | 13.3 | 11.1 | 16.1 | 10.1 | 1.20 | 0.91 to 1.58 |
| Emergence time | Shen 2014 | 60 | 60 | 13.07 | 9.20 | 6.56 | 4.40 | 1.38 | 1.15 to 1.65 |
|  | Ohkushi 2016 [S] | 20 | 20 | 8.21 | 14.35 | 3.30 | 11.20 | 0.54 | 0.37 to 0.79 |
|  | Chung 2018 | 50 | 50 | 2.5 | 2.9 | 1.0 | 0.9 | 1.26 | 0.75 to 0.99 |
|  | Castillo 2014 | 29 | 30 | 17.0 | 9.0 | 7.0 | 3.0 | 2.29 | 1.56 to 2.29 |
|  | Shah 2018 | 25 | 25 | 8.0 | 3.4 | 0.7 | 1.2 | 2.35 | 2.04 to 2.71 |
|  | Mustapure 2024 | 25 | 25 | 8.5 | 11.4 | 2.3 | 2.7 | 1.15 | 0.65 to 0.86 |
|  | Kumar 2025 | 55 | 55 | 6.1 | 8.3 | 1.8 | 2.2 | 1.13 | 0.66 to 0.82 |
|  | Lakshmi 2025 | 32 | 32 | 7.5 | 6.2 | 1.6 | 1.4 | 1.21 | 1.09 to 1.35 |
|  | Kim 2020 | 50 | 49 | 4.9 | 7.4 | 1.3 | 1.3 | 1.06 | 0.61 to 0.72 |
|  | Parida 2014 [S] | 50 | 50 | 7.0 | 8.0 | 3.0 | 4.0 | 1.28 | 0.73 to 1.05 |
|  | Kowark 2018 [S] | 118 | 111 | 9.8 | 10.1 | 5.1 | 4.0 | 1.37 | 0.86 to 1.09 |

Ratios of means (ROM) were computed as Mean_TIVA_/Mean_Sevo_ on the log scale to account for the log-normal distribution of time-to-event recovery outcomes (PACU length of stay and emergence time). A ROM <1.0 indicates a faster mean recovery time with propofol TIVA, whereas a ROM >1.0 indicates a faster mean recovery time with sevoflurane. [S] denotes studies included exclusively in the sensitivity analyses (all eligible cohort) based on pre-specified methodological criteria.

CI: confidence interval; PACU: post-anesthesia care unit; ROM: ratio of means; SD: standard deviation; Sevo: sevoflurane; TIVA: total intravenous anesthesia.

# Table S6. Study-level ratios of standard deviations (RSD) for continuous recovery time endpoints

**Part A: Core Studies**

| Outcome | Study ID | RSD (SD_TIVA_/SD_Sevo_) | ln(RSD) | SE ln(RSD) | 95% CI |
| --- | --- | --- | --- | --- | --- |
| PACU length of stay | Kim 2020 | 1.23 | −0.18 | 0.14 | 0.63 to 1.10 |
|  | Mustapure 2024 | 1.25 | −0.16 | 0.20 | 0.57 to 1.27 |
|  | Kumar 2025 | 1.17 | −0.26 | 0.14 | 0.59 to 1.01 |
|  | Lakshmi 2025 | 1.07 | 0.06 | 0.18 | 0.75 to 1.52 |
| Emergence time | Shen 2014 | 1.49 | 0.40 | 0.13 | 1.16 to 1.92 |
|  | Chung 2018 | 1.11 | 0.11 | 0.14 | 0.84 to 1.47 |
|  | Castillo 2014 | 2.33 | 1.25 | 0.19 | 1.62 to 3.37 |
|  | Shah 2018 | 0.58 | −0.54 | 0.20 | 0.39 to 0.87 |
|  | Mustapure 2024 | 1.25 | −0.16 | 0.20 | 0.57 to 1.27 |
|  | Kumar 2025 | 1.22 | −0.20 | 0.14 | 0.63 to 1.07 |
|  | Lakshmi 2025 | 1.14 | 0.13 | 0.18 | 0.80 to 1.63 |
|  | Kim 2020 | 1.00 | 0.00 | 0.14 | 0.75 to 1.33 |

**Part B: Sensitivity Analysis (All Eligible Studies)**

| Outcome | Study ID | RSD (SD_TIVA_/SD_Sevo_) | ln(RSD) | SE ln(RSD) | 95% CI |
| --- | --- | --- | --- | --- | --- |
| PACU length of stay | Kim 2020 | 1.23 | −0.18 | 0.14 | 0.63 to 1.10 |
|  | Mustapure 2024 | 1.25 | −0.16 | 0.20 | 0.57 to 1.27 |
|  | Kumar 2025 | 1.17 | −0.26 | 0.14 | 0.59 to 1.01 |
|  | Lakshmi 2025 | 1.07 | 0.06 | 0.18 | 0.75 to 1.52 |
|  | Kowark 2018 [S] | 1.59 | 0.47 | 0.09 | 1.33 to 1.92 |
| Emergence time | Shen 2014 | 1.49 | 0.40 | 0.13 | 1.16 to 1.92 |
|  | Ohkushi 2016 [S] | 0.31 | −1.19 | 0.23 | 0.19 to 0.48 |
|  | Chung 2018 | 1.11 | 0.11 | 0.14 | 0.84 to 1.47 |
|  | Castillo 2014 | 2.33 | 1.25 | 0.19 | 1.62 to 3.37 |
|  | Shah 2018 | 0.58 | −0.54 | 0.20 | 0.39 to 0.87 |
|  | Mustapure 2024 | 1.25 | −0.16 | 0.20 | 0.57 to 1.27 |
|  | Kumar 2025 | 1.22 | −0.20 | 0.14 | 0.63 to 1.07 |
|  | Lakshmi 2025 | 1.14 | 0.13 | 0.18 | 0.80 to 1.63 |
|  | Kim 2020 | 1.00 | 0.00 | 0.14 | 0.75 to 1.33 |
|  | Parida 2014 [S] | 1.15 | −0.29 | 0.14 | 0.57 to 0.99 |
|  | Kowark 2018 [S] | 1.27 | 0.24 | 0.09 | 1.06 to 1.53 |

The Ratio of Standard Deviations (RSD) was computed as (SD_TIVA_/SD_Sevo_) on the log scale to evaluate differences in between-patient recovery variability between techniques. An RSD < 1.0 indicates lower variability (more predictable recovery times) with propofol TIVA, whereas an RSD > 1.0 indicates lower variability with sevoflurane. [S] designates studies included exclusively in the sensitivity analysis cohort.

CI: confidence interval; PACU: post-anesthesia care unit; RSD: ratio of standard deviations; SD: standard deviation; SE: standard error; Sevo: sevoflurane; TIVA: total intravenous anesthesia.

# Figure S1. Forest plot: PACU length of stay.

# Figure S2. Forest plot: emergence time.

# Figure S3. Contour-enhanced funnel plot: PONV incidence.

# Figure S4. Trim-and-fill funnel plot: PONV incidence.

# Figure S5. Forest plot: rescue antiemetics administration.

# Figure S6. Sensitivity analysis: leave-one-out analysis for PONV incidence.

# Figure S7. Influence analysis for PONV incidence.

# Figure S8. Forest plot: subgroup analysis on PONV incidence by TIVA administration mode.

# Figure S9. Meta-regression: effect of publication year on PONV RR.

# Figure S10. Meta-regression: effect of sample size on PONV RR.

# Figure S11. Cumulative meta-analysis of PONV incidence.

# Figure S12. PONV incidence in core and sensitivity studies.

# Figure S13. PACU length of stay, ratio of means.

# Figure S14. Emergence time, ratio of means.

# Supplementary references

[1]	Jiang JL, Zhang L, He LL, et al. Volatile Versus Total Intravenous Anesthesia on Postoperative Delirium in Adult Patients Undergoing Cardiac Valve Surgery: A Randomized Clinical Trial. Anesth Analg 2023;136:60–9. https://doi.org/10.1213/ANE.0000000000006257.

[2]	Zhang Y, Shan GJ, Zhang YX, et al. Propofol compared with sevoflurane general anaesthesia is associated with decreased delayed neurocognitive recovery in older adults. Br J Anaesth 2018;121:595–604. https://doi.org/10.1016/j.bja.2018.05.059.

[3]	Nieuwenhuijs-Moeke GJ, Nieuwenhuijs VB, Seelen MAJ, et al. Propofol-based anaesthesia versus sevoflurane-based anaesthesia for living donor kidney transplantation: results of the VAPOR-1 randomized controlled trial. Br J Anaesth 2017;118:720–32. https://doi.org/10.1093/bja/aex057.

[4]	Lindholm EE, Aune E, Frøland G, et al. Analysis of transthoracic echocardiographic data in major vascular surgery from a prospective randomised trial comparing sevoflurane and fentanyl with propofol and remifentanil anaesthesia. Anaesthesia 2014;69:558–72. https://doi.org/10.1111/anae.12604.

[5]	Wang G, Liu JIE, Gao J, et al. Comparison of the effects of sevoflurane and propofol anesthesia on pulmonary function, MMP-9 and postoperative cognition in patients receiving lung cancer resection. Oncol Lett 2019;17:3399–405. https://doi.org/10.3892/ol.2019.9993.

[6]	Yao J, Gao Z, Qu W, et al. Propofol total intravenous anesthesia vs. sevoflurane inhalation anesthesia: Effects on post‑operative cognitive dysfunction and inflammation in geriatric patients undergoing laparoscopic surgery. Exp Ther Med 2024;28. https://doi.org/10.3892/etm.2024.12632.

[7]	Cao SJ, Zhang Y, Zhang YX, et al. Delirium in older patients given propofol or sevoflurane anaesthesia for major cancer surgery: a multicentre randomised trial. Br J Anaesth 2023;131:253–65. https://doi.org/10.1016/j.bja.2023.04.024.

[8]	Franzén S, Semenas E, Taavo M, et al. Renal function during sevoflurane or total intravenous propofol anaesthesia: a single-centre parallel randomised controlled study. Br J Anaesth 2022;128:838–48. https://doi.org/10.1016/j.bja.2022.02.030.

[9]	Goswami U, Babbar S, Tiwari S. Comparative evaluation of the effects of propofol and sevoflurane on cognitive function and memory in patients undergoing laparoscopic cholecystectomy: A randomised prospective study. Indian J Anaesth 2015;59:150–5. https://doi.org/10.4103/0019-5049.153036.

[10]	Munmany M, Gracia M, Nonell R, et al. The use of inhaled sevoflurane during operative hysteroscopy is associated with increased glycine absorption compared to intravenous propofol for maintenance of anesthesia. J Clin Anesth 2016;31:202–7. https://doi.org/10.1016/j.jclinane.2016.02.012.

[11]	Study Details | CTRI/2022/05/042869 | To compare the effect of total intravenous anaesthesia and inhalation anaesthesia on postoperative quality of recovery in patients undergoing open pelvic organ surgery- a randomised controlled trial | Clinical Trials Registry India n.d. https://ctri.nic.in/Clinicaltrials/pmaindet2.php?EncHid=Njg3MTE=&Enc=&userName= (accessed February 19, 2026).

[12]	Study Details | NCT04793022 | Total Intravenous Anesthesia With Propofol vs. General Anesthesia in Outpatient Shoulder Arthroscopic Surgeries | ClinicalTrials.gov n.d. https://clinicaltrials.gov/study/NCT04793022 (accessed February 19, 2026).

[13]	Na SH, Jeong KH, Eum D, et al. Patient quality of recovery on the day of surgery after propofol total intravenous anesthesia for vitrectomy: A randomized controlled trial. Medicine 2018;97. https://doi.org/10.1097/MD.0000000000012699.

[14]	Oliveros H, Ríos F, Botero-Rosas DA, et al. Variability of anesthetic depth in total intravenous anesthesia vs balanced anesthesia using entropy indices: A randomized, crossover, controlled clinical trial. Colombian Journal of Anesthesiology 2020;48:111–7. https://doi.org/10.1097/CJ9.0000000000000163.

[15]	Jones KA, LaFerriere KA. Intravenous Anesthesia With Bispectral Index Monitoring vs Inhalational Anesthesia for Rhytidoplasty: A Randomized Clinical Trial. JAMA Facial Plast Surg 2015;17:239–44. https://doi.org/10.1001/jamafacial.2015.0222.

[16]	Prathep S, Mahattanaporn S, Wasinwong W. Target Controlled Infusion versus Sevoflurane/Desflurane Anesthesia for Laparoscopic Cholecystectomy: Comparison Postoperative Nausea/Vomiting and Extubation Time. J Med Assoc Thai 2015;98:1187–92.

[17]	Ding F, Wang X, Zhang L, et al. Effect of propofol-based total intravenous anaesthesia on postoperative cognitive function and sleep quality in elderly patients. Int J Clin Pract 2021;75. https://doi.org/10.1111/ijcp.14266.

[18]	Study Details | NCT02272166 | Effects of Propofol on Early Recovery of Hunger After Surgery | ClinicalTrials.gov n.d. https://clinicaltrials.gov/study/NCT02272166 (accessed February 19, 2026).

[19]	Saylan S, Cobanoglu HBY. Effect of classical laryngeal mask or I-gel use on otolaryngeal system in ambulatory inhalation general anesthesia: A prospective study. Pak J Med Sci 2024;40:2299–304. https://doi.org/10.12669/pjms.40.10.8416.

[20]	Kapoor PM, Taneja S, Kiran U, et al. Comparison of the effects of inhalational anesthesia with desflurane and total intravenous anesthesia on cardiac biomarkers after aortic valve replacement. Ann Card Anaesth 2015;18:502–9. https://doi.org/10.4103/0971-9784.166455.

[21]	Kampmeier T, Rehberg S, Omar Alsaleh AJ, et al. Cost-Effectiveness of Propofol (Diprivan) Versus Inhalational Anesthetics to Maintain General Anesthesia in Noncardiac Surgery in the United States. Value Health 2021;24:939–47. https://doi.org/10.1016/j.jval.2021.01.008.

[22]	Piri E, Purtuloglu T, Guler L, et al. Comparison of Propofol and Desflurane Anesthesia Combined with Remifentanyl in Breast Surgery. Journal of Clinical and Analytical Medicine 2015;6:144–8.

[23]	Miller TE, Gan TJ. Total intravenous anesthesia and anesthetic outcomes. J Cardiothorac Vasc Anesth 2015;29 Suppl 1:S11–5. https://doi.org/10.1053/j.jvca.2015.01.022.

[24]	Saravanan B, Kundra P, Mishra SK, et al. Effect of anaesthetic agents on olfactory threshold and identification - A single blinded randomised controlled study. Indian J Anaesth 2018;62:592–8. https://doi.org/10.4103/ija.IJA_245_18.

[25]	Study Details | NCT04307238 | Effects of Different Types of General Anesthesia on Postoperative Pupillary Reactivity | ClinicalTrials.gov n.d. https://clinicaltrials.gov/study/NCT04307238 (accessed February 19, 2026).

[26]	Baxter MG, Mincer JS, Brallier JW, et al. Cognitive Recovery by Decade in Healthy 40- to 80-Year-Old Volunteers After Anesthesia Without Surgery. Anesth Analg 2022;134:389–99. https://doi.org/10.1213/ANE.0000000000005824.

[27]	Lasala J, Mena GE, Iniesta MD, et al. Impact of anesthesia technique on post-operative opioid use in open gynecologic surgery in an enhanced recovery after surgery pathway. Int J Gynecol Cancer 2021;31:569–74. https://doi.org/10.1136/ijgc-2020-002004.

[28]	Shi Y, Wang W. Application of different anesthetic methods in coronary artery bypass grafting and the effect on postoperative outcome. Exp Ther Med 2019;17. https://doi.org/10.3892/etm.2018.6993.

[29]	Attri JP, Singh M, Bhatt H, et al. Application of Discharge Criteria for Home Readiness Using Bispectral and Supraglottic Airway Devices in Day-care Surgery without Using Muscle Relaxants. Anesth Essays Res 2017;11:816. https://doi.org/10.4103/aer.aer_20_17.

[30]	Adis Medical Writers. Balance risks and benefits when selecting anaesthetic and perioperative care options in elderly surgical outpatients. Drugs & Therapy Perspectives 2018 34:3 2018;34:121–7. https://doi.org/10.1007/s40267-018-0478-2.

[31]	Abstracts of Posters presented at the International Anesthesia Research Society 2019 Annual Meeting and International Science Symposium. Anesth Analg 2019;128:1–999. https://doi.org/10.1213/01.ane.0000721552.99259.00.

[32]	Clinical Trials register - Search for Changes in the human brain induced by general anaesthesia. n.d. https://www.clinicaltrialsregister.eu/ctr-search/search?query=Changes+in+the+human+brain+induced+by+general+anaesthesia. (accessed February 19, 2026).

[33]	Study Details | NCT03441828 | Effects of Depth of Neuromuscular Block on Surgical Operating Conditions in Gynecologic Laparoscopic Surgery | ClinicalTrials.gov n.d. https://clinicaltrials.gov/study/NCT03441828 (accessed February 19, 2026).

[34]	Vaughan J, Nagendran M, Cooper J, et al. Anaesthetic regimens for day-procedure laparoscopic cholecystectomy. Cochrane Database Syst Rev 2014;2014. https://doi.org/10.1002/14651858.CD009784.pub2.

[35]	Shu Z, Lin T, Xu D, et al. Comparison of sevoflurane and propofol in combination with remifentanil on the quality of postoperative recovery in patients undergoing laparoscopic bariatric surgery. Front Med (Lausanne) 2024;11. https://doi.org/10.3389/fmed.2024.1382724.

[36]	Besir A, Cekic B, Kutanis D, et al. Comparison of surgical conditions in 2 different anesthesia techniques of esmolol-induced controlled hypotension in breast reduction surgery. Medicine 2017;96. https://doi.org/10.1097/MD.0000000000006254.
