# Supplementary material for: Recovery Kinetics and Postoperative Outcomes of Propofol‐Based Total Intravenous Anesthesia Versus Sevoflurane in Ambulatory Surgery: A Systematic Review and Meta‐Analysis of Randomized Studies
Source: Anesthesiol Res Pract. 2026 Oct 1;2026:2531080. doi: 10.1155/anrp/2531080 (PMC13628567; doi:10.1155/anrp/2531080)
Supplement: Supplementary file 1 [file ANRP-2026-2531080-s001.zip › Table S2.docx]

# **Table S2. Major exclusion with reason for exclusion.**

Of 51 full-text articles assessed for eligibility, 36 were excluded with reasons as follows:

| **Reason for exclusion** | **n** |
| --- | --- |
| Wrong setting (inpatient/major surgery, cardiac surgery) | 10 |
| Not an RCT (review articles, retrospective studies, protocols) | 8 |
| Wrong comparator (desflurane only, no sevoflurane arm) | 4 |
| Missing relevant outcomes | 5 |
| Duplicate publication | 1 |
| Other (wrong intervention, trial registration only, etc.) | 8 |
| Total | 36 |

**List of excluded studies (with reasons)**

| **#** | **Citation (Author, Year, Title)** | **Reason for exclusion** |
| --- | --- | --- |
| [1] | Jiang J et al. 2023. Volatile Versus Total Intravenous Anesthesia on Postoperative Delirium in Adult Patients Undergoing Cardiac Valve Surgery: A Randomized Clinical Trial. | Wrong setting (cardiac surgery). |
| [2] | Zhang Y et al. 2018. Propofol compared with sevoflurane general anaesthesia is associated with decreased delayed neurocognitive recovery in older adults. | Wrong setting (inpatient, major surgery). |
| [3] | Nieuwenhuijs-Moeke G et al. 2017. Propofol-based anaesthesia versus sevoflurane-based anaesthesia for living donor kidney transplantation: results of the VAPOR-1 randomized controlled trial. | Wrong setting (inpatient). |
| [4] | Lindholm E et al. 2014. Analysis of transthoracic echocardiographic data in major vascular surgery from a prospective randomised trial comparing sevoflurane and fentanyl with propofol and remifentanil anaesthesia. | Wrong setting (major vascular surgery); no relevant outcomes measured. |
| [5] | Wang G et al. 2019. Comparison of the effects of sevoflurane and propofol anesthesia on pulmonary function, MMP-9 and postoperative cognition in patients receiving lung cancer resection. | Wrong setting (inpatient); no relevant outcomes. |
| [6] | Yao J et al. 2024. Propofol total intravenous anesthesia vs. sevoflurane inhalation anesthesia: Effects on post-operative cognitive dysfunction and inflammation in geriatric patients undergoing laparoscopic surgery. | Not randomized. |
| [7] | Cao S et al. 2023. Delirium in older patients given propofol or sevoflurane anaesthesia for major cancer surgery: a multicentre randomised trial. | Wrong setting (major surgery). |
| [8] | Franzen S et al. 2022. Renal function during sevoflurane or total intravenous propofol anaesthesia: a single-centre parallel randomised controlled study. | Wrong setting (inpatient); renal outcomes only (no relevant recovery outcomes). |
| [9] | Goswami U et al. 2015. Comparative evaluation of the effects of propofol and sevoflurane on cognitive function and memory in patients undergoing laparoscopic cholecystectomy: A randomised prospective study. | Missing relevant outcomes (no discharge readiness/PACU LOS/emergence time/PONV with suitable numerics). |
| [10] | Munmany M et al. 2016. The use of inhaled sevoflurane during operative hysteroscopy is associated with increased glycine absorption compared to intravenous propofol for maintenance of anesthesia. | Wrong setting (inpatient); wrong outcome (glycine absorption). |
| [11] | CTRI/2022/05/042869. 2022. Quality of recovery in patients undergoing open pelvic surgery under general anaesthesia using total intravenous anaesthesia or full inhalation anaesthesia. | Trial registration only (no full report). |
| [12] | NCT04793022. 2021. Total Intravenous Anesthesia With Propofol vs. General Anesthesia in Outpatient Shoulder Arthroscopic Surgeries. | Trial registration only (no full report). |
| [13] | Na SH et al. 2018. Patient quality of recovery on the day of surgery after propofol total intravenous anesthesia for vitrectomy: a randomized controlled trial. | Wrong comparator (desflurane). |
| [14] | Oliveros H et al. 2020. Variability of anesthetic depth in total intravenous anesthesia vs balanced anesthesia using entropy indices: a randomized, crossover, controlled clinical trial. | Missing relevant outcomes (entropy/narrative outcomes only). |
| [15] | Jones KA et al. 2015. Intravenous Anesthesia With Bispectral Index Monitoring vs Inhalational Anesthesia for Rhytidoplasty: a Randomized Clinical Trial. | Review/not meeting eligibility (pregled). |
| [16] | Prathep S et al. 2015. Target Controlled Infusion versus Sevoflurane/Desflurane Anesthesia for Laparoscopic Cholecystectomy: comparison Postoperative Nausea/Vomiting and Extubation Time. | Not RCT; no appropriate comparator. |
| [17] | Ding F et al. 2021. Effect of propofol-based total intravenous anaesthesia on postoperative cognitive function and sleep quality in elderly patients. | Wrong setting (inpatient). |
| [18] | Effects of Propofol on Early Recovery of Hunger After Surgery (NCT02272166 / Besnier). 2020. | Duplicate publication (duplicate of Besnier 2020). |
| [19] | Saylan S et al. 2024. Effect of classical laryngeal mask or I-gel use on otolaryngeal system in ambulatory inhalation general anesthesia: A prospective study. | Wrong comparison (I-gel vs LMA, not propofol vs sevoflurane). |
| [20] | Kapoor P et al. 2015. Comparison of the effects of inhalational anesthesia with desflurane and total intravenous anesthesia on cardiac biomarkers after aortic valve replacement. | Wrong comparator (desflurane); wrong setting (cardiac). |
| [21] | Kampmeier T et al. 2021. Cost-Effectiveness of Propofol (Diprivan) Versus Inhalational Anesthetics to Maintain General Anesthesia in Noncardiac Surgery in the United States. | Narrative/cost-effectiveness (not RCT of recovery outcomes). |
| [22] | Piri E et al. 2015. Comparison of Propofol and Desflurane Anesthesia Combined with Remifentanyl in Breast Surgery. | Wrong comparator (desflurane only). |
| [23] | Miller TE et al. 2015. Total Intravenous Anesthesia and Anesthetic Outcomes. | Review article. |
| [24] | Saravanan B et al. 2018. Effect of anaesthetic agents on olfactory threshold and identification - A single blinded randomised controlled study. | Missing relevant outcomes; short procedures not explicit day-case (excluded from quantitative meta-analysis). |
| [25] | NCT04307238. 2019. Effects of Different Types of General Anesthesia on Postoperative Pupillary Reactivity. | Trial registration only (no full report). |
| [26] | Baxter M et al. 2022. Cognitive Recovery by Decade in Healthy 40- to 80-Year-Old Volunteers After Anesthesia Without Surgery. | Wrong intervention (no TIVA comparison as per inclusion). |
| [27] | Lasala J et al. 2020. Impact of anesthesia technique on post-operative opioid use in open gynecologic surgery in an enhanced recovery after surgery pathway. | Retrospective study (not RCT). |
| [28] | Shi Y et al. 2019. Application of different anesthetic methods in coronary artery bypass grafting and the effect on postoperative outcome. | Wrong setting (CABG / cardiac surgery). |
| [29] | Attri J et al. 2017. Application of Discharge Criteria for Home Readiness Using Bispectral and Supraglottic Airway Devices in Day-care Surgery without Using Muscle Relaxants. | Wrong comparator (desflurane). |
| [30] | Adis Medical Writers. 2018. Balance risks and benefits when selecting anaesthetic and perioperative care options in elderly surgical outpatients. Drugs Ther Perspect. | Review article. |
| [31] | No author. 2019. International Anesthesia Research Society 2019 Annual Meeting and International Science Symposium. Anesth Analg. | Abstract book / conference (not full article). |
| [32] | EUCTR2018-001252-35-DK. 2018. Changes in the human brain induced by general anaesthesia. | Review / not full trial report. |
| [33] | NCT03441828. 2018. Effects of Depth of Neuromuscular Block on Surgical Operating Conditions in Gynecologic Laparoscopic Surgery. | Wrong topic (neuromuscular block; not propofol vs sevoflurane). |
| [34] | Vaughan J et al. Anaesthetic regimens for day-procedure laparoscopic cholecystectomy. | Review article. |
| [35] | Shu et al. 2024. Comparison of sevoflurane and propofol in combination with remifentanil on the quality of postoperative recovery in patients undergoing laparoscopic bariatric surgery. | Missing relevant outcomes (no outcome measured as required). |
| [36] | Besir A et al. 2017. Comparison of surgical conditions in 2 different anesthesia techniques of esmolol-induced controlled hypotension in breast reduction surgery. | After verification: wrong comparison (esmolol-induced hypotension / surgical conditions; not propofol vs sevoflurane for recovery outcomes). |

Total: 36 (matches protocol).

Note:

This table was populated from the screening file fulltext_retrieval_wnakon pyton screen - fulltext_retrieval_with_rct_status is.csv (column F = continues after individual full-text screening, 0 = excluded, reason in G; column H = continues after full-text reading, 0 = excluded, reason in I). Reasons are given as in the source; some were translated or standardized for PRISMA (wrong setting, not RCT, wrong comparator, missing outcomes, duplicate, other).

**Exclusion reason categories (use consistently):**

Wrong setting — Inpatient, major surgery, cardiac surgery, or non-ambulatory setting

Not RCT — Review, retrospective study, protocol without results, or non-randomized design

Wrong comparator — Desflurane (or other volatile) only, no sevoflurane arm

Missing outcomes — No relevant recovery/ambulatory outcomes reported

Duplicate — Duplicate publication of an already included study

Other — Wrong intervention (e.g. propofol induction only), trial registration only, or other (specify in table)
