# Supplementary material for: Recovery Kinetics and Postoperative Outcomes of Propofol‐Based Total Intravenous Anesthesia Versus Sevoflurane in Ambulatory Surgery: A Systematic Review and Meta‐Analysis of Randomized Studies
Source: Anesthesiol Res Pract. 2026 Oct 1;2026:2531080. doi: 10.1155/anrp/2531080 (PMC13628567; doi:10.1155/anrp/2531080)
Supplement: Supplementary file 1 [file ANRP-2026-2531080-s001.zip › Table S3.docx]

# **Table S3. Characteristics of the four studies included in the sensitivity assessment.**

| Study | Type of study | Country | N. patients total | N. TIVA | N. Sevoflurane | Surgery Type | Setting | ASA risk class | TIVA Mode | Antiemetic Prophylaxis |
| --- | --- | --- | --- | --- | --- | --- | --- | --- | --- | --- |
| Parida S 2014 | RCT | NR | 100 | 50 | 50 | Mixed day-care surgery | Day Surgery | NR | NR | NR |
| Ohkushi K 2016 | Cross-over study | Japan | 20 | 20 | 20 | Dental surgery | Day Surgery | 1-2 | TCI | NR |
| Kowark A 2018 | RCT | Germany | 343 | 118 | 111 (+114 desflurane) | Mixed day-care surgery | Day Surgery | NR | TCI | NR |
| Cablay KJ 2024 | RCT | USA | 82 | 42 | 40 | Shoulder arthroscopy | Day Surgery | NR | TCI | NR |

RCT: randomized controlled trial; NR: not reported; TCI: Target Controlled Infusion; ASA: American Society of Anesthesiology
