# Supplementary material for: Recovery Kinetics and Postoperative Outcomes of Propofol‐Based Total Intravenous Anesthesia Versus Sevoflurane in Ambulatory Surgery: A Systematic Review and Meta‐Analysis of Randomized Studies
Source: Anesthesiol Res Pract. 2026 Oct 1;2026:2531080. doi: 10.1155/anrp/2531080 (PMC13628567; doi:10.1155/anrp/2531080)
Supplement: Supplementary file 1 [file ANRP-2026-2531080-s001.zip › Table S4.docx]

# **Table S4. Trim-and-fill sensitivity analysis for publication bias (PONV incidence).**

| Parameter | Value |
| --- | --- |
| Outcome evaluated | PONV incidence |
| k | 9 |
| Imputed studies (theoretical missing trials) | 5 |
| k_adj_ | 14 |
| Adjusted pooled RR | 0.78 |
| 95% CI | 0.55 to 1.10 |
| Adjusted p-value | 0.138 |
| Interpretation | The 95% CI spans the null value (1.0); the adjusted estimate moves toward the null, indicating that small-study effects attenuate the observed antiemetic benefit of TIVA. |

PONV: postoperative nausea and vomiting; k: studies in core analysis; k_adj_: total studies after trim-and-fill; RR: risk ratio; CI: confidence interval; TIVA: total intravenous anesthesia.
