# Supplementary material for: Recovery Kinetics and Postoperative Outcomes of Propofol‐Based Total Intravenous Anesthesia Versus Sevoflurane in Ambulatory Surgery: A Systematic Review and Meta‐Analysis of Randomized Studies
Source: Anesthesiol Res Pract. 2026 Oct 1;2026:2531080. doi: 10.1155/anrp/2531080 (PMC13628567; doi:10.1155/anrp/2531080)
Supplement: Supplementary file 1 [file ANRP-2026-2531080-s001.zip › Table S5.docx]

# **Table S5. Study-level ratios of means for recovery times (PACU length of stay and emergence time)**

**Part A: Core Studies**

| Outcome | Study ID | N. (TIVA) | N. (Sevo) | Mean TIVA (min) | Mean Sevo (min) | SD TIVA | SD Sevo | ROM | 95% CI |
| --- | --- | --- | --- | --- | --- | --- | --- | --- | --- |
| PACU length of stay | Kim 2020 | 50 | 49 | 15.7 | 17.0 | 3.0 | 3.6 | 1.32 | 0.85 to 1.00 |
|  | Mustapure 2024 | 25 | 25 | 35.4 | 40.5 | 10.2 | 12.0 | 1.27 | 0.74 to 1.03 |
|  | Kumar 2025 | 55 | 55 | 11.4 | 14.8 | 2.7 | 3.5 | 1.17 | 0.71 to 0.84 |
|  | Lakshmi 2025 | 32 | 32 | 29.5 | 25.2 | 8.0 | 7.5 | 1.17 | 1.02 to 1.35 |
| Emergence time | Shen 2014 | 60 | 60 | 13.07 | 9.20 | 6.56 | 4.40 | 1.38 | 1.15 to 1.65 |
|  | Chung 2018 | 50 | 50 | 2.5 | 2.9 | 1.0 | 0.9 | 1.26 | 0.75 to 0.99 |
|  | Castillo 2014 | 29 | 30 | 17.0 | 9.0 | 7.0 | 3.0 | 2.29 | 1.56 to 2.29 |
|  | Shah 2018 | 25 | 25 | 8.0 | 3.4 | 0.7 | 1.2 | 2.35 | 2.04 to 2.71 |
|  | Mustapure 2024 | 25 | 25 | 8.5 | 11.4 | 2.3 | 2.7 | 1.15 | 0.65 to 0.86 |
|  | Kumar 2025 | 55 | 55 | 6.1 | 8.3 | 1.8 | 2.2 | 1.13 | 0.66 to 0.82 |
|  | Lakshmi 2025 | 32 | 32 | 7.5 | 6.2 | 1.6 | 1.4 | 1.21 | 1.09 to 1.35 |
|  | Kim 2020 | 50 | 49 | 4.9 | 7.4 | 1.3 | 1.3 | 1.06 | 0.61 to 0.72 |

**Part B: Sensitivity Analysis (All Eligible Studies)**

| Outcome | Study ID | N (TIVA) | N (Sevo) | Mean TIVA (min) | Mean Sevo (min) | SD TIVA | SD Sevo | ROM | 95% CI |
| --- | --- | --- | --- | --- | --- | --- | --- | --- | --- |
| PACU length of stay | Kim 2020 | 50 | 49 | 15.7 | 17.0 | 3.0 | 3.6 | 1.32 | 0.85 to 1.00 |
|  | Mustapure 2024 | 25 | 25 | 35.4 | 40.5 | 10.2 | 12.0 | 1.27 | 0.74 to 1.03 |
|  | Kumar 2025 | 55 | 55 | 11.4 | 14.8 | 2.7 | 3.5 | 1.17 | 0.71 to 0.84 |
|  | Lakshmi 2025 | 32 | 32 | 29.5 | 25.2 | 8.0 | 7.5 | 1.17 | 1.02 to 1.35 |
|  | Kowark 2018 [S] | 118 | 111 | 13.3 | 11.1 | 16.1 | 10.1 | 1.20 | 0.91 to 1.58 |
| Emergence time | Shen 2014 | 60 | 60 | 13.07 | 9.20 | 6.56 | 4.40 | 1.38 | 1.15 to 1.65 |
|  | Ohkushi 2016 [S] | 20 | 20 | 8.21 | 14.35 | 3.30 | 11.20 | 0.54 | 0.37 to 0.79 |
|  | Chung 2018 | 50 | 50 | 2.5 | 2.9 | 1.0 | 0.9 | 1.26 | 0.75 to 0.99 |
|  | Castillo 2014 | 29 | 30 | 17.0 | 9.0 | 7.0 | 3.0 | 2.29 | 1.56 to 2.29 |
|  | Shah 2018 | 25 | 25 | 8.0 | 3.4 | 0.7 | 1.2 | 2.35 | 2.04 to 2.71 |
|  | Mustapure 2024 | 25 | 25 | 8.5 | 11.4 | 2.3 | 2.7 | 1.15 | 0.65 to 0.86 |
|  | Kumar 2025 | 55 | 55 | 6.1 | 8.3 | 1.8 | 2.2 | 1.13 | 0.66 to 0.82 |
|  | Lakshmi 2025 | 32 | 32 | 7.5 | 6.2 | 1.6 | 1.4 | 1.21 | 1.09 to 1.35 |
|  | Kim 2020 | 50 | 49 | 4.9 | 7.4 | 1.3 | 1.3 | 1.06 | 0.61 to 0.72 |
|  | Parida 2014 [S] | 50 | 50 | 7.0 | 8.0 | 3.0 | 4.0 | 1.28 | 0.73 to 1.05 |
|  | Kowark 2018 [S] | 118 | 111 | 9.8 | 10.1 | 5.1 | 4.0 | 1.37 | 0.86 to 1.09 |

Ratios of means (ROM) were computed as Mean_TIVA_/Mean_Sevo_ on the log scale to account for the log-normal distribution of time-to-event recovery outcomes (PACU length of stay and emergence time). A ROM <1.0 indicates a faster mean recovery time with propofol TIVA, whereas a ROM >1.0 indicates a faster mean recovery time with sevoflurane. [S] denotes studies included exclusively in the sensitivity analyses (all eligible cohort) based on pre-specified methodological criteria.

CI: confidence interval; PACU: post-anesthesia care unit; ROM: ratio of means; SD: standard deviation; Sevo: sevoflurane; TIVA: total intravenous anesthesia.
