# Supplementary material for: Recovery Kinetics and Postoperative Outcomes of Propofol‐Based Total Intravenous Anesthesia Versus Sevoflurane in Ambulatory Surgery: A Systematic Review and Meta‐Analysis of Randomized Studies
Source: Anesthesiol Res Pract. 2026 Oct 1;2026:2531080. doi: 10.1155/anrp/2531080 (PMC13628567; doi:10.1155/anrp/2531080)
Supplement: Supplementary file 1 [file ANRP-2026-2531080-s001.zip › Table S6.docx]

# **Table S6. Study-level ratios of standard deviations (RSD) for continuous recovery time endpoints**

**Part A: Core Studies**

| Outcome | Study ID | RSD (SD_TIVA_/SD_Sevo_) | ln(RSD) | SE ln(RSD) | 95% CI |
| --- | --- | --- | --- | --- | --- |
| PACU length of stay | Kim 2020 | 1.23 | −0.18 | 0.14 | 0.63 to 1.10 |
|  | Mustapure 2024 | 1.25 | −0.16 | 0.20 | 0.57 to 1.27 |
|  | Kumar 2025 | 1.17 | −0.26 | 0.14 | 0.59 to 1.01 |
|  | Lakshmi 2025 | 1.07 | 0.06 | 0.18 | 0.75 to 1.52 |
| Emergence time | Shen 2014 | 1.49 | 0.40 | 0.13 | 1.16 to 1.92 |
|  | Chung 2018 | 1.11 | 0.11 | 0.14 | 0.84 to 1.47 |
|  | Castillo 2014 | 2.33 | 1.25 | 0.19 | 1.62 to 3.37 |
|  | Shah 2018 | 0.58 | −0.54 | 0.20 | 0.39 to 0.87 |
|  | Mustapure 2024 | 1.25 | −0.16 | 0.20 | 0.57 to 1.27 |
|  | Kumar 2025 | 1.22 | −0.20 | 0.14 | 0.63 to 1.07 |
|  | Lakshmi 2025 | 1.14 | 0.13 | 0.18 | 0.80 to 1.63 |
|  | Kim 2020 | 1.00 | 0.00 | 0.14 | 0.75 to 1.33 |

**Part B: Sensitivity Analysis (All Eligible Studies)**

| Outcome | Study ID | RSD (SD_TIVA_/SD_Sevo_) | ln(RSD) | SE ln(RSD) | 95% CI |
| --- | --- | --- | --- | --- | --- |
| PACU length of stay | Kim 2020 | 1.23 | −0.18 | 0.14 | 0.63 to 1.10 |
|  | Mustapure 2024 | 1.25 | −0.16 | 0.20 | 0.57 to 1.27 |
|  | Kumar 2025 | 1.17 | −0.26 | 0.14 | 0.59 to 1.01 |
|  | Lakshmi 2025 | 1.07 | 0.06 | 0.18 | 0.75 to 1.52 |
|  | Kowark 2018 [S] | 1.59 | 0.47 | 0.09 | 1.33 to 1.92 |
| Emergence time | Shen 2014 | 1.49 | 0.40 | 0.13 | 1.16 to 1.92 |
|  | Ohkushi 2016 [S] | 0.31 | −1.19 | 0.23 | 0.19 to 0.48 |
|  | Chung 2018 | 1.11 | 0.11 | 0.14 | 0.84 to 1.47 |
|  | Castillo 2014 | 2.33 | 1.25 | 0.19 | 1.62 to 3.37 |
|  | Shah 2018 | 0.58 | −0.54 | 0.20 | 0.39 to 0.87 |
|  | Mustapure 2024 | 1.25 | −0.16 | 0.20 | 0.57 to 1.27 |
|  | Kumar 2025 | 1.22 | −0.20 | 0.14 | 0.63 to 1.07 |
|  | Lakshmi 2025 | 1.14 | 0.13 | 0.18 | 0.80 to 1.63 |
|  | Kim 2020 | 1.00 | 0.00 | 0.14 | 0.75 to 1.33 |
|  | Parida 2014 [S] | 1.15 | −0.29 | 0.14 | 0.57 to 0.99 |
|  | Kowark 2018 [S] | 1.27 | 0.24 | 0.09 | 1.06 to 1.53 |

The Ratio of Standard Deviations (RSD) was computed as (SD_TIVA_/SD_Sevo_) on the log scale to evaluate differences in between-patient recovery variability between techniques. An RSD < 1.0 indicates lower variability (more predictable recovery times) with propofol TIVA, whereas an RSD > 1.0 indicates lower variability with sevoflurane. [S] designates studies included exclusively in the sensitivity analysis cohort.

CI: confidence interval; PACU: post-anesthesia care unit; RSD: ratio of standard deviations; SD: standard deviation; SE: standard error; Sevo: sevoflurane; TIVA: total intravenous anesthesia.
